# Development of sub-tropically adapted diverse provitamin-A rich maize inbreds through marker-assisted pedigree selection, their characterization and utilization in hybrid breeding

**DOI:** 10.1371/journal.pone.0245497

**Published:** 2021-02-04

**Authors:** Hriipulou Duo, Firoz Hossain, Vignesh Muthusamy, Rajkumar U. Zunjare, Rajat Goswami, Gulab Chand, Subhra J. Mishra, Rashmi Chhabra, Munegowda M. Gowda, Saikat Pal, Aanchal Baveja, Jayant S. Bhat, Mehar C. Kamboj, Bhupender Kumar, John J. Amalraj, Rajesh Khulbe, Bhukya Prakash, C. N. Neeraja, Sujay Rakshit, Om P. Yadav

**Affiliations:** 1 Division of Genetics, ICAR-Indian Agricultural Research Institute, New Delhi, India; 2 CCS-Haryana Agricultural University, Regional Research Station, Uchani, Haryana, India; 3 ICAR-Indian Institute Maize Research, Ludhiana, Punjab, India; 4 Tamil Nadu Agricultural University, Coimbatore, Tamil Nadu, India; 5 ICAR-Vivekananda Parvatiya Krishi Anusandhan Sansthan, Almora, India; 6 ICAR-Directorate of Poultry Research, Hyderabad, Telangana State, India; 7 ICAR-Indian Institute of Rice Research, Hyderabad, Telangana State, India; Faculty of Agriculture (FoA), Sher-e-Kashmir University of Agricultural Sciences and Technology of Kashmir (SKUAST-K), Wadura Campus, INDIA

## Abstract

Malnutrition has emerged as one of the major health problems worldwide. Traditional yellow maize has low provitamin-A (proA) content and its genetic base in proA biofortification breeding program of subtropics is extremely narrow. To diversify the proA rich germplasm, 10 elite low proA inbreds were crossed with a proA rich donor (HP702-22) having mutant *crtRB1* gene. The F_2_ populations derived from these crosses were genotyped using InDel marker specific to *crtRB1*. Severe marker segregation distortion was observed. Seventeen *crtRB1* inbreds developed through marker-assisted pedigree breeding and seven inbreds generated using marker-assisted backcross breeding were characterized using 77 SSRs. Wide variation in gene diversity (0.08 to 0.79) and dissimilarity coefficient (0.28 to 0.84) was observed. The inbreds were grouped into three major clusters depicting the existing genetic diversity. The *crtRB1*-based inbreds possessed high β-carotene (BC: 8.72μg/g), β-cryptoxanthin (BCX: 4.58μg/g) and proA (11.01μg/g), while it was 2.35μg/g, 1.24μg/g and 2.97μg/g in checks, respectively. Based on their genetic relationships, 15 newly developed *crtRB1*-based inbreds were crossed with five testers (having *crtRB1* gene) using line × tester mating design. 75 experimental hybrids with *crtRB1* gene were evaluated over three locations. These experimental hybrids possessed higher BC (8.02μg/g), BCX (4.69μg/g), proA (10.37μg/g) compared to traditional hybrids used as check (BC: 2.36 μg/g, BCX: 1.53μg/g, proA: 3.13μg/g). Environment and genotypes × environment interaction had minor effects on proA content. Both additive and dominance gene action were significant for proA. The mean proportion of proA to total carotenoids (TC) was 44% among *crtRB1*-based hybrids, while 11% in traditional hybrids. BC was found to be positively correlated with BCX (r = 0.68) and proA (r = 0.98). However, no correlation was observed between proA and grain yield. Several hybrids with >10.0 t/ha grain yield with proA content >10.0 μg/g were identified. This is the first comprehensive study on development of diverse proA rich maize hybrids through marker-assisted pedigree breeding approach. The findings provides sustainable and cost-effective solution to alleviate vitamin-A deficiency.

## Introduction

Malnutrition due to consumption of unbalanced diet affects two billion people worldwide [1]. Deficiency of micronutrients in the long run causes severe socio-economic problems. Among micronutrients, vitamin-A deficiency (VAD) is one of the major health problems found to subsist in human population [2]. Vitamin-A is essential for vision, immunity and various metabolisms [3]. Night blindness and complete loss of vision are the hallmarks of the VAD in humans [4]. The deficiency also induces higher risk to severe infections such as measles, diarrhoea and weakened immunity among children and pregnant women [5]. While 30% of preschool-age children and more than 19 million pregnant women in developing countries are vitamin-A deficient, 5.2 million of the same age preschool-age groups and 9.7 million pregnant women suffer from clinically night blindness (www.harvestplus.org).

Various avenues namely, food-fortification, medical-supplementation and food-diversification are implemented to alleviate VAD [6,7]. However, sustainability of these approaches is limited by the weak distribution system, low purchasing power of the rural people and crop seasonality [8]. ‘Crop-biofortification’ where micronutrient density is enhanced in edible parts of food through plant breeding, has now emerged as the most popular choice to address malnutrition through cost-efficient and sustainable approach [9]. Biofortified staple crops when consumed regularly have been found to improve the human health [10,11].

Maize (*Zea mays* L.) is an important cereal crop grown in almost all parts of the world and cultivated across diverse climatic spectrum [12]. It is a source of food to billions of people and also used as feed for poultry and livestock [13]. Traditional yellow kernel maize possesses high kernel carotenoids, but composed predominantly of non-provitamin-A (non-proA) fractions [lutein (LUT) and zeaxanthin (ZEA)] and very less provitamin-A (proA) fraction [α-carotene and β-carotene (BC), β-cryptoxanthin (BCX)] [14,15]. ProA content in traditional tropical maize is quite low (0.25–2.50 μg/g) and far-off from the targeted concentration of 15 μg/g as set by HarvestPlus [1]. *CrtRB1* gene that codes for *β-carotene hydroxylase* is associated with higher accumulation of proA especially BC and BCX in maize. Rare natural variation in *crtRB1* gene limits the hydroxylation of BC and BCX [16]. The wild type allele possesses a transposable element (TE) in 3’UTR region of the *crtRB1*, while the TE is absent in mutant version [17].

Diverse proA rich inbreds have been developed in the tropics [17–19]. However, the genetic base of proA rich inbreds is extremely low in the sub-tropical regions and thus the frequency of favourable *crtRB1* allele is extremely low in Indian maize germplasm [2,20]. Therefore, strengthening the breeding programme by broadening the genetic base of proA germplasm assumes great significance. The present study was, therefore undertaken to (i) develop sub-tropically adapted diverse *crtRB1*-inbreds through marker-assisted pedigree breeding, (ii) characterize the newly developed *crtRB1*-based inbreds using microsatellite markers, (iii) study combining ability of the *crtRB1*-based inbreds for different carotenoid fractions, and (iv) identify promising hybrids with high proA and grain yield.

## Materials and methods

### Development of *crtRB1*-based inbreds

#### Genetic materials

Ten elite normal maize inbreds, *viz*., UMI-1200, UMI-1230, BML-6Q, BML-7Q, LM-11Q, LM-12Q, LM-13Q, LM-14Q, PDM-4341 and PDM-4251, possessing good general combining ability for greater yield but low in proA were selected as recipient parents. To incorporate favourable allele of *crtRB1* into these inbreds, a CIMMYT-HarvestPlus bred inbred i.e., HP704-22 was used as donor. All these recipient inbreds represent a great extent of adaptation range as parental lines of released/promising hybrids while the donor HP704-22 has poor adaptation in Indian conditions (S1 Table). Crosses were made during rainy season (July-October) of 2015 at the ICAR-Indian Agricultural Research Institute (ICAR-IARI), New Delhi (29°41´52.13ʺN and 77°0´24.95ʺE). F_1_s were raised and selfed during winter season (December-April) of 2015–16 at the Winter Nursery Centre (WNC), ICAR-Indian Institute of Maize Research (ICAR-IIMR), Hyderabad (17°21´50.39ʺN and 78°29´42.31ʺE). Ten F_2_ populations consisting of 99–111 plants of each cross were then grown at ICAR-IARI, New Delhi during rainy season (2016) (Table 1).

**Table 1:** Segregation pattern of *crtRB1* gene in F_2_ populations across the crosses.

S. No.	Cross combination	N	CC	C^+^C	C^+^C^+^	df	*χ*^2^
1.	UMI-1200 × HP704-22	105	23	37	45	2	18.37**
2.	UMI-1230 × HP704-22	101	19	46	36	2	6.52*
3.	BML-6Q × HP704-22	99	22	24	53	2	45.69**
4.	BML-7Q × HP704-22	106	20	41	45	2	17.23**
5.	LM-11Q × HP704-22	106	20	33	53	2	35.64**
6.	LM-12Q × HP704-22	105	15	48	42	2	14.87**
7.	LM-13Q × HP704-22	108	27	40	41	2	10.89**
8.	LM-14Q × HP704-22	102	21	41	40	2	11.00**
9.	PDM-4341 × HP704-22	100	16	46	38	2	10.32*
10.	PDM-4251 × HP704-22	111	18	51	42	2	11.11**
Total		1043	201	407	435	2	155.27**

**^,^* Significant at P = 0.01, 0.05, respectively N = No. of plants genotyped, df = degrees of freedom, C^+^: unfavourable allele of *crtRB1*, C: favourable allele of *crtRB1*.

#### Genotyping of F_2_ populations

The F_1_s were tested for hybridity using *crtRB1*-based *InDel* marker present in 3’UTR. The true F_1_s were selfed to derive F_2_ populations. Genomic DNA was extracted from three weeks old seedlings of F_2_ progenies using standard procedure of CTAB with minor modification [21]. The quality of genomic DNA was checked on 0.8% agarose gel and quantified on UV-spectrophotometer (BT-UVS-SBA-E, G-Biosciences). Genotyping was carried out using *3’TE-InDel*-based marker for *crtRB1* [16], *viz*., Forward (F): 5’ACACCACATGGACAAGTTCG3’, Reverse1 (R1):5’ACACTCTGGCCCATGAACAC3’ and Reverse2 (R2): 5’ACAGCAATACAGGGGACCAG3’. The primers were custom synthesized from Macrogen Inc., Seoul, South Korea. Polymerase chain reaction (PCR) protocol for *crtRB1* [22] standardized at Maize Genetics Unit, ICAR-IARI, New Delhi was further modified for reducing the time duration. *In-vitro* amplification using ready-to-use master mix OnePCR^TM^ (GeneDireX, Inc.) including *Taq* buffer, MgCl_2_, dNTPs and *Taq* Polymerase was used to perform PCR reaction in 96 well microtiter plate (M/s Genaxy) using GenePro thermal cycler (M/s Hangzhou Bioer Technology Co. Ltd.). The amplified product was resolved on 1.5% Seakem LE agarose gel (LONZA, Rockland, USA).

#### Advancement of desirable *crtRB1* homozygotes

The F_2_ segregants with homozygous *crtRB1* were selfed and derived F_3_ progenies were raised at ICAR-IARI, New Delhi during rainy season (2017). The F_4_s were raised during winter season (2017–18) at WNC, ICAR-IIMR, Hyderabad, and F_5_ progenies during rainy season (2018) at ICAR-IARI, New Delhi. To avoid chances of out-crossing, the presence of favourable allele of *crtRB1* in each generation was validated using *crtRB1*-based *InDel* marker. Desirable segregants were advanced to further generation based on plant-, ear- and grain- characteristics.

### Characterization of inbreds

#### Genetic materials

Fifteen *crtRB1*-based inbreds (MGU-PVMAS-1 to MGU-PVMAS-15) from 10 F_2_ populations were selected for molecular characterization (S2 Table). The donor line (HP704-22) used for introgression of *crtRB1* was also included. Marker-assisted pedigree breeding was used to derive these 16 inbreds. Besides, seven proA lines (PMI-PV-1, PMI-PV-2, PMI-PV-5, PMI-PV-6, PMI-PV-7, PMI-PV-8 and PMI-PV-9) earlier developed by marker-assisted backcross breeding (MABB) at ICAR-IARI were also included. PMI-PV-1 and PMI-PV-2 are parental inbreds of India’s first proA rich maize hybrid (Pusa Vivek QPM9 Improved). PMI-PV-5, PMI-PV-6, PMI-PV-7, PMI-PV-8 and PMI-PV-9 are the parents of MABB-derived proA rich hybrids (APQH-1, APQH-4, Pusa HQPM5 Improved, Pusa HQPM7 Improved and APQH-8). Further, HP465-41, a CIMMYT-derived proA rich inbred derived through marker-assisted pedigree breeding was also included for characterization. Two low proA elite inbreds (PMI-Q2 and PMI-Q3, possessing the unfavourable allele of *crtRB1*) were also included as control for quality analysis. These 26 inbreds were planted in randomized complete block design (RCBD) at ICAR-IARI, New Delhi in rainy season of 2018. Each inbred was planted with two replications, in rows of 3 m with a plant-to-plant distance of 20 cm. The rows were spaced 75 cm apart. Recommended cultural practices were followed to raise a good experimental crop. To avoid contamination by foreign pollens, 2–3 plants in each row were selfed for estimation of carotenoids.

#### DNA extraction and PCR

Selfed seeds of 24 inbreds with favourable allele of *crtRB1* were used for molecular analysis using simple sequence repeats (SSRs) markers. DNA was isolated from seeds using standard sodium dodecyl sulphate (SDS) extraction protocol [23]. A total of 77 SSRs distributed across the genome were used for characterization. The information of SSR markers with bin locations and nature of SSR repeats at each locus are provided in Table 2. Primer sequence information of maize SSRs was retrieved from public domain (MaizeGDB; http://www.maizegdb.org). PCR was carried out as per Choudhary et al. (2016) [24]. The PCR amplified products for each SSR were resolved on 4% agarose gel stained with 0.4 mg/ml ethidium bromide using horizontal electrophoresis system at 120 V for 3–4 h.

**Table 2:** Primer details and summary statistics of genotyping assay in 24 inbreds.

S. No.	Marker	Bin	Repeats	Major allele Frequency	Number of alleles	Gene Diversity	Hetero-zygosity	PIC
1	*bnlg1866*	1.03	(AG)_11_	0.46	3.00	0.64	0.08	0.57
2	*umc1770*	1.04	(GGC)_4_	0.92	2.00	0.15	0.00	0.14
3	*umc1833*	1.07	(TG)_8_	0.67	3.00	0.47	0.08	0.39
4	*umc1446*	1.08	(TAA)_7_	0.60	5.00	0.59	0.25	0.55
5	*umc2240*	1.08	(AC)_6_	0.71	3.00	0.45	0.00	0.41
6	*umc2223*	1.10	(GCG)_4_	0.88	2.00	0.22	0.00	0.19
7	*umc1737*	1.11	(AGA)_7_	0.79	3.00	0.35	0.04	0.32
8	*phi064*	1.11	ATCC	0.33	6.00	0.77	0.13	0.74
9	*umc2244*	1.12	(GGC)_4_	0.67	3.00	0.50	0.08	0.45
10	*umc2246*	2.00	(CCTCCT)_4_	0.71	3.00	0.43	0.00	0.37
11	*umc1227*	2.01	(AGG)_4_	0.67	2.00	0.44	0.00	0.35
12	*umc1448*	2.01	(GCT)_5_	0.56	4.00	0.61	0.08	0.57
13	*phi126*	2.02	AG	0.29	6.00	0.78	0.17	0.74
14	*umc2193*	2.02	(TCC)_6_	0.48	3.00	0.62	0.04	0.55
15	*bnlg1537*	2.03	(AG)_16_	0.44	4.00	0.69	0.13	0.63
16	*bnlg1396*	2.06	(AG)_15_	0.83	3.00	0.29	0.00	0.27
17	*umc1912*	2.06	(GCG)_6_	0.83	2.00	0.28	0.00	0.24
18	*umc1057*	3.02	(CGG)_6_	0.88	2.00	0.22	0.00	0.19
19	*bnlg1325*	3.03	(AG)_18_	0.38	6.00	0.77	0.04	0.74
20	*bnlg1523*	3.03	(AG)_17_	0.42	4.00	0.71	0.04	0.66
21	*phi029*	3.04	AG/AGCG***	0.46	3.00	0.62	0.00	0.54
22	*umc2261*	3.04	(GAAGAG)_4_	0.77	3.00	0.38	0.04	0.34
23	*bnlg1638*	3.04	(AG)_25_	0.40	6.00	0.76	0.04	0.73
24	*mmc0071*	3.05	(GA)_21_	0.63	4.00	0.53	0.08	0.46
25	*umc2267*	3.06	(CTTG)_5_	0.63	3.00	0.52	0.00	0.44
26	*umc1844*	3.08	(TC)_8_	0.71	4.00	0.47	0.00	0.43
27	*umc1010*	3.09	(GA)_10_	0.46	3.00	0.62	0.00	0.54
28	*umc1136*	3.10	(GCA)_5_	0.48	3.00	0.60	0.04	0.51
29	*umc1294*	4.02	(GAG)_4_	0.44	4.00	0.67	0.04	0.61
30	*bnlg1162*	4.03	(AG)_21_	0.40	6.00	0.75	0.04	0.71
31	*umc2061*	4.05	(CTG)_8_	0.38	3.00	0.66	0.00	0.59
32	*bnlg252*	4.06	-	0.83	3.00	0.29	0.00	0.26
33	*phi093*	4.08	AGCT	0.52	3.00	0.57	0.00	0.48
34	*umc1173*	4.09	(AC)_7_	0.54	2.00	0.50	0.00	0.37
35	*umc2139*	4.09	(GCC)_4_	0.54	3.00	0.53	0.08	0.43
36	*umc2044*	4.10	(CGG)_6_	0.44	3.00	0.61	0.08	0.53
37	*umc1761*	5.02	(GCA)_5_	0.46	4.00	0.67	0.13	0.61
38	*umc2167*	5.03	(CGC)_6_	0.96	2.00	0.08	0.00	0.08
39	*umc2298*	5.04	(GCG)_4_	0.79	2.00	0.33	0.00	0.28
40	*umc1060*	5.04	(CGG)_5_	0.54	2.00	0.50	0.00	0.37
41	*umc1941*	5.06	(CTG)_10_	0.81	3.00	0.32	0.08	0.30
42	*bnlg1346*	5.07	(AG)_24_	0.40	5.00	0.72	0.25	0.67
43	*umc2308*	5.08	(CGGCG)_4_	0.58	2.00	0.49	0.00	0.37
44	*umc1792*	5.08	(CGG)_5_	0.50	3.00	0.57	0.00	0.48
45	*umc1153*	5.09	(TCA)_4_	0.71	3.00	0.45	0.08	0.40
46	*bnlg249*	6.01	-	0.63	4.00	0.54	0.00	0.48
47	*umc1257*	6.02	(CAC)_4_	0.75	2.00	0.38	0.00	0.30
48	*umc1006*	6.02	(GA)_19_	0.75	3.00	0.41	0.00	0.37
49	*umc1857*	6.04	(TAA)_6_	0.83	2.00	0.29	0.00	0.25
50	*bnlg2249*	6.05	(AG)_20_	0.88	2.00	0.22	0.00	0.19
51	*bnlg1922*	6.05	(AG)_17_	0.46	3.00	0.62	0.00	0.54
52	*bnlg1740*	6.07	(AG)_21_	0.31	6.00	0.79	0.17	0.75
53	*umc2325*	7.01	(TGG)_7_	0.46	3.00	0.64	0.00	0.57
54	*umc1409*	7.01	(GCTC)_4_	0.85	2.00	0.25	0.13	0.22
55	*umc1068*	7.02	(GAAA)_6_(GAA)_2_	0.96	2.00	0.08	0.00	0.08
56	*bnlg1022*	7.02	(AG)_12_	0.58	5.00	0.61	0.00	0.57
57	*umc1929*	7.02	(GA)_10_	0.58	2.00	0.49	0.00	0.37
58	*umc1112*	7.03	(TC_)6_	0.81	2.00	0.30	0.04	0.26
59	*umc1242*	7.05	(TAA)_6_	0.54	2.00	0.50	0.00	0.37
60	*umc2190*	7.06	(CCT)_4_	0.46	4.00	0.62	0.08	0.55
61	*phi119*	8.02	AG	0.63	4.00	0.52	0.13	0.45
62	*umc1802*	8.03	(CA)_8_	0.58	2.00	0.49	0.00	0.37
63	*bnlg240*	8.06	-	0.63	4.00	0.56	0.00	0.52
64	*bnlg1272*	9.0	(AG)_16_	0.37	5.00	0.75	0.13	0.71
65	*phi028*	9.01	GAA	0.71	2.00	0.41	0.00	0.33
66	*umc1370*	9.01	(CGGG)_5_	0.92	3.00	0.16	0.04	0.15
67	*umc1170*	9.02	(TC)_12_	0.78	3.00	0.36	0.00	0.32
68	*umc2099*	9.07	(ATGC)_5_	0.75	2.00	0.38	0.00	0.30
69	*umc1318*	10.01	(GTC)_5_	0.56	2.00	0.49	0.04	0.37
70	*umc1152*	10.02	(ATAG)_6_	0.58	2.00	0.49	0.00	0.37
71	*umc2180*	10.03	(GGCC)_4_	0.58	4.00	0.57	0.00	0.50
72	*umc1381*	10.03	(AAC)_4_	0.88	2.00	0.22	0.00	0.19
73	*bnlg210*	10.03	-	0.65	3.00	0.52	0.08	0.47
74	*umc1678*	10.04	(TCG)_6_	0.69	4.00	0.47	0.08	0.42
75	*umc1898*	10.05	(CGC)_4_	0.48	4.00	0.61	0.04	0.54
76	*umc2122*	10.06	(TG)_8_	0.46	5.00	0.64	0.00	0.57
77	*umc2172*	10.07	(ATCC)_5_	0.77	3.00	0.38	0.04	0.35
Mean				0.62	3.23	0.49	0.04	0.43

PIC: Polymorphism Information Content

*** repeat length variable.

#### Genetic diversity analysis

For each allele, presence of a band in a genotype was indicated by 1 and absence of the band as 0. Five parameters, *viz*., gene diversity, major allele frequency, total number of alleles detected, heterozygosity and polymorphism information content (PIC) were estimated using PowerMarker v3.0 [25]. An allele appearing only in one genotype was scored as unique allele, while an allele with a frequency of ≤0.05 was considered as a rare allele. Genetic dissimilarity analysis using Jaccard’s coefficient was calculated and tree was constructed using Neighbour-Joining (NJ) pattern in DARwin-6.0 [26]. Principal coordinate analysis (PCoA) was also carried out to complement the clustering pattern [27].

#### Estimation of carotenoids from inbreds

Carotenoids from the selfed seeds of 26 inbreds (24 with favourable allele of *crtRB1* and two inbreds with unfavourable allele of *crtRB1*) were extracted from maize endosperm through protocol of Kurilich and Juvik (1999) [28] with modifications. Carotenoids were quantified using Dionex Ultimate 3000 UHPLC System (Ultra High-Performance Liquid Chromatography; Thermo Scientific, Massachusetts, USA). Samples were eluted through YMC Carotenoid C_30_ column (5 μm, 4.6 × 250 mm; YMC) and detected with a diode array detector-3000 (RS). The mobile phase comprised of methanol: tert-butyl methyl ether (80:20, v/v) with flow rate at 1 ml/min and peaks were detected at 450 nm. For each carotenoid component, *viz*., BC, BCX, LUT and ZEA, six dilutions of standards were used to construct the regression curve. To estimate proA concentration, amount of BC was added to one-half of BCX amount, while sum of LUT and ZEA gave the non-proA fractions [15]. Total carotenoid (TC) was calculated by adding the value of BC, BCX, LUT and ZEA [29].

### Hybrids evaluation

#### Combining ability analysis

A set of 15 *crtRB1*-based inbreds developed under the current study were crossed with five *crtRB1*-based tester inbreds (PMI-PV-5, PMI-PV-6, PMI-PV-7, PMI-PV-9 and HP465-41) as per line × tester mating design [30] at WNC, Hyderabad during winter season (2017–18) to generate 75 hybrid combinations. While, PMI-PV-5, PMI-PV-6, PMI-PV-7 and PMI-PV-9 are the parents of proA rich elite hybrids developed earlier through MABB, HP465-41 is a CIMMYT-derived *crtRB1*-based promising inbred. These five testers belonged to two different groups viz., -A (PMI-PV-5 and HP465-41), -B (PMI-PV-6, PMI-PV-7 and PMI-PV-9). The 75 hybrid combinations and five commercial check hybrids (CoMH-08-292 and DHM-121: low in proA; and ‘Pusa HQPM5 Improved’, ‘Pusa HQPM7 Improved’ and ‘Pusa Vivek QPM9 Improved’: high in proA) were evaluated using RCBD at three locations, *viz*. ICAR-IARI, New Delhi; CCS-HAU Regional Station, Uchani (29°68^’^ N, 76°99^’^ E, 255 MSL); and ICAR-IARI Regional Research Centre, Dharwad (15°45^’^ N, 75°0078^’^ E, 750 MSL) in rainy season of 2018. Each entry was evaluated in two replications, and was grown in a single row of 3 m length, with a row-to-row distance of 75 cm and plant-to-plant distance of 20 cm. Combining ability of the inbreds for carotenoids was calculated as per Singh and Choudhary (1985) [31].

#### Estimation of carotenoids from hybrids

In each of the 75 experimental hybrid combinations generated from crosses, 2–3 plants were selfed to avoid contamination by the foreign pollen. The selfed seeds were used for estimation of carotenoids using UHPLC as per the procedure depicted for the inbreds.

#### Heterosis for grain yield

Grain yield (YLD) per plot was converted to t/ha as per the standard procedure. Magnitude of heterosis in hybrids over five commercial checks was estimated following Singh and Choudhary (1985) [31].

#### Statistical analysis

The statistical analyses on ANOVA, correlation coefficients and combining ability were computed using Windostat 8.0.

## Results

### Selection of *crtRB1*-based segregants in F_2_ populations

**Fig 1:**
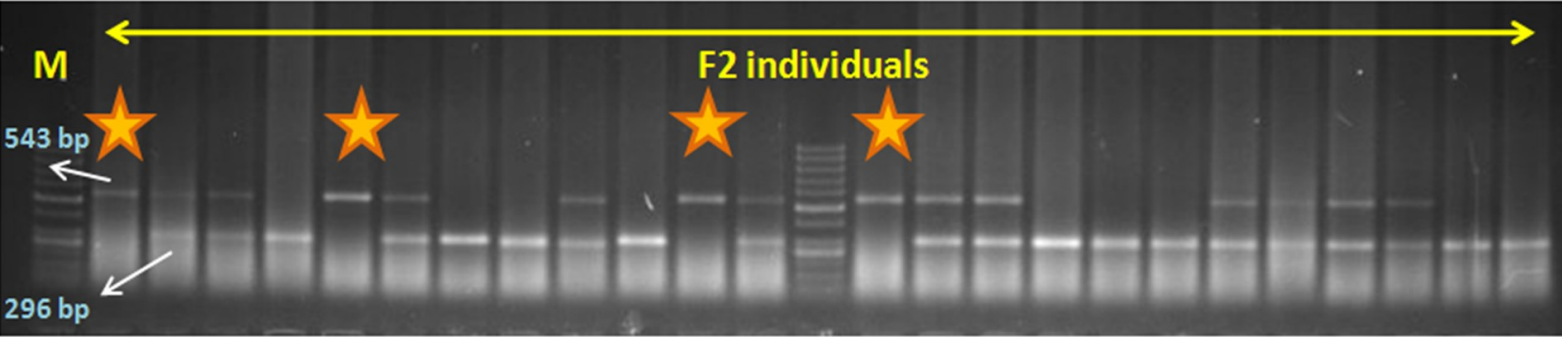
Segregation of favourable (543 bp) and unfavourable (296 bp) alleles of *crtRB1* in F_2_ populations. Star indicates favourable homozygous individuals.

The recipient parents produced an amplicon of 296 bp, while the donor, a 543 bp amplicon. The true F_1_s had both 296 and 543 bp amplicons. The 10 F_2_ populations were genotyped using *crtRB1*-specific *3’TE-InDel*-based marker (Table 1). A representative gel depicting the segregation of *crtRB1* gene in F_2_s is presented in Fig 1. Of the total 1043 segregants genotyped, only 201 were homozygous for favourable allele of*crtRB1*. The heterozygotes and homozygotes (wild-type allele) were 407 and 435, respectively. Thus, *crtRB1* showed severe marker segregation distortion both cumulatively as well as in individual populations. Out of 201 favourable homozygotes, 75 segregants were selected based on ear- and grain- characteristics, and advanced to generate F_3_ progenies. Finally, 15 locally adapted F_4_ progenies (S2 Table) representing all 10 crosses were selected for further characterization.

### Characterization of inbreds using microsatellite markers

**Fig 2:**
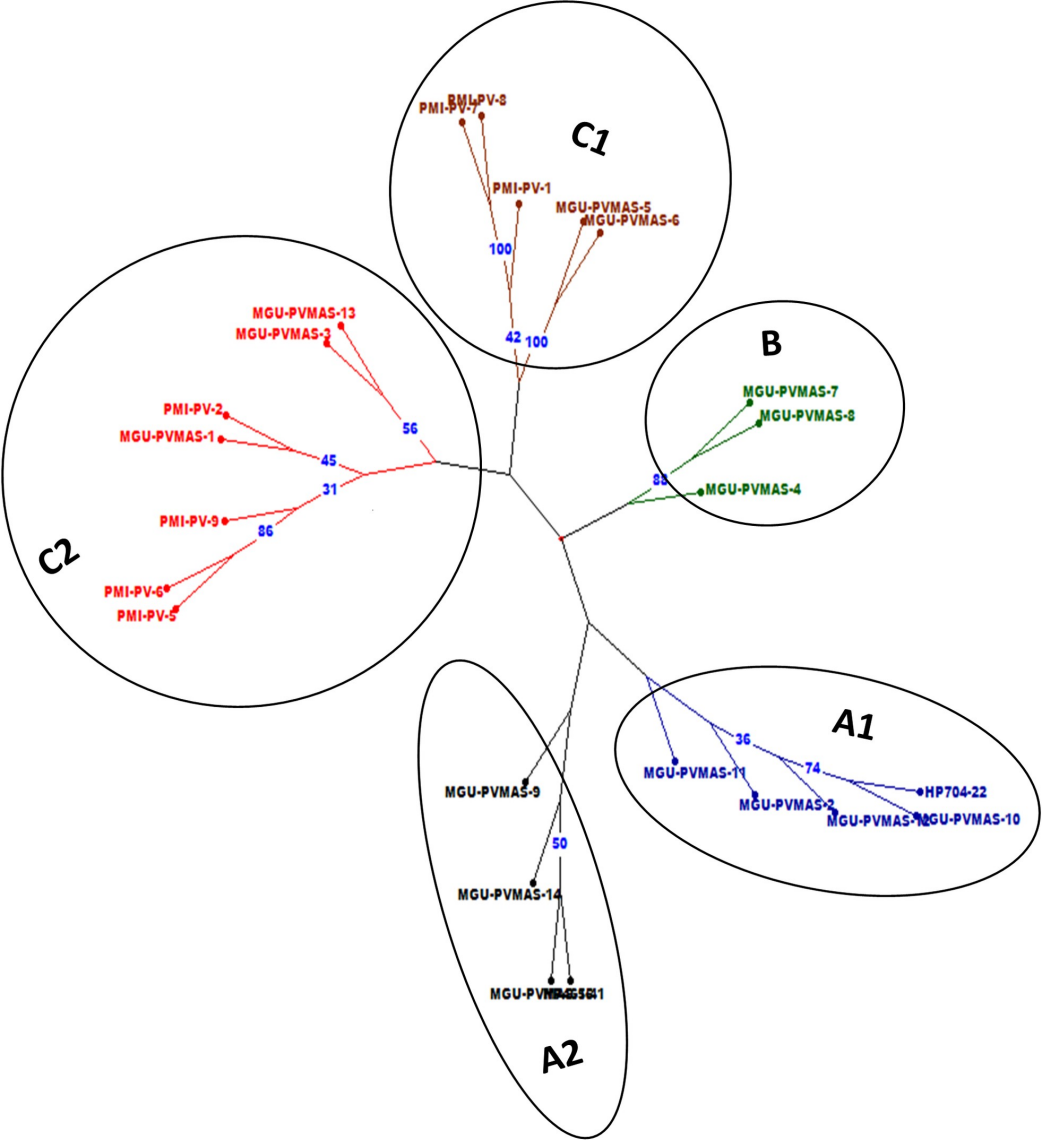
Cluster analysis depicting genetic dissimilarity of the 24 inbreds. Three major clusters viz., A, B and C while 1 and 2 represent sub-clusters within each major cluster. Bootstrap value ≥30 is presented.

**Fig 3:**
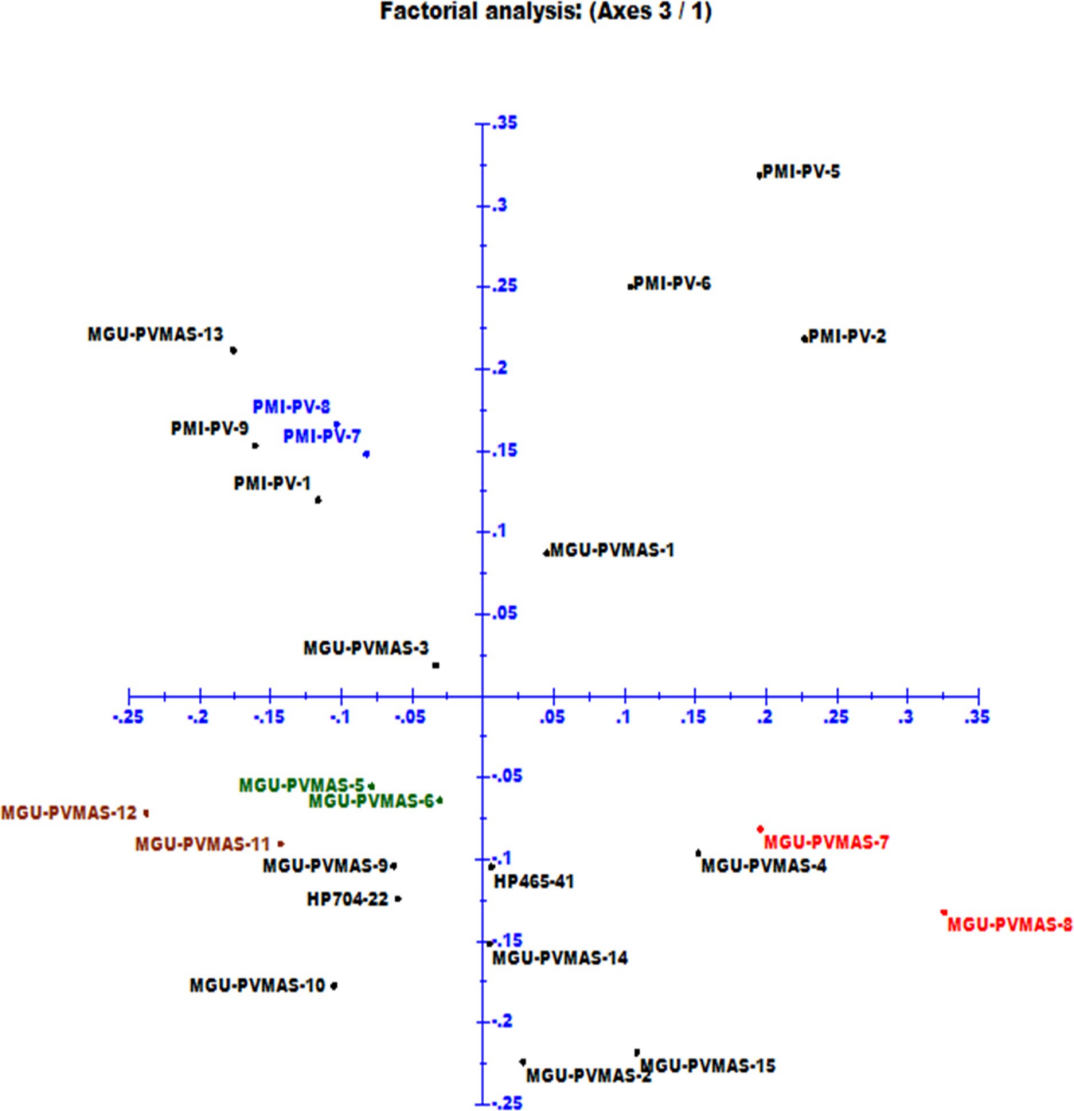
Principle coordinate analysis (PCoA) among 24 proA inbreds using SSRs.

Characterization of 24 *crtRB1*-based inbreds including 15 new inbreds developed under the study using 77 SSRs showed existence of 249 alleles, with mean of 3.23 and range of 2–6 alleles per SSR locus (Table 2). 24, 28, 14, 5 and 6 loci revealed two, three, four, five and 6 alleles, respectively among the *crtRB1*-based inbreds. Amplified allele size ranged from 45 bp (*bnlg1162*) to 270 bp (*bnlg1537*). The average major allele frequency was 0.62 with a range from 0.29 (*phi126*) to 0.96 (*umc2167* and *umc1068*). A set of 24 SSRs showed major allele frequency of ≤0.5. The average gene diversity was 0.49, ranging from 0.08 (*umc2167* and *umc1068*) to 0.79 (*bnlg1740*) (Table 2). The PIC score of markers ranged from 0.08 (*umc2167* and *umc1068*) to 0.75 (*bnlg1740*) with an average of 0.43. Of the 77 SSRs, 29 loci had PIC ≥0.5. The current study also detected 14 unique alleles and 23 rare alleles. The heterozygosity existing among the inbreds varied from 0.00 to 0.25 with a mean of 0.04.Some loci such as *umc1446* (0.25) and *bnlg1346* (0.25) showed high heterozygosity (Table 2). Genetic dissimilarities assessed among 24 *crtRB1*-based inbreds through cluster analysis showed a range from 0.28 (MGU-PVMAS-5 and MGU-PVMAS-6) to 0.84 (MGU-PVMAS-13 and MGU-PVMAS-8) with mean of 0.67 (S3 Table). Cluster analysis grouped the inbreds, including lines and testers used in crosses, into three major clusters namely, -A, -B and -C (Fig 2). PCoA distributed the 24 inbreds to four quadrangles (Fig 3).

### Genetic variability for kernel carotenoids

#### Variation of carotenoids among inbreds

**Fig 4:**
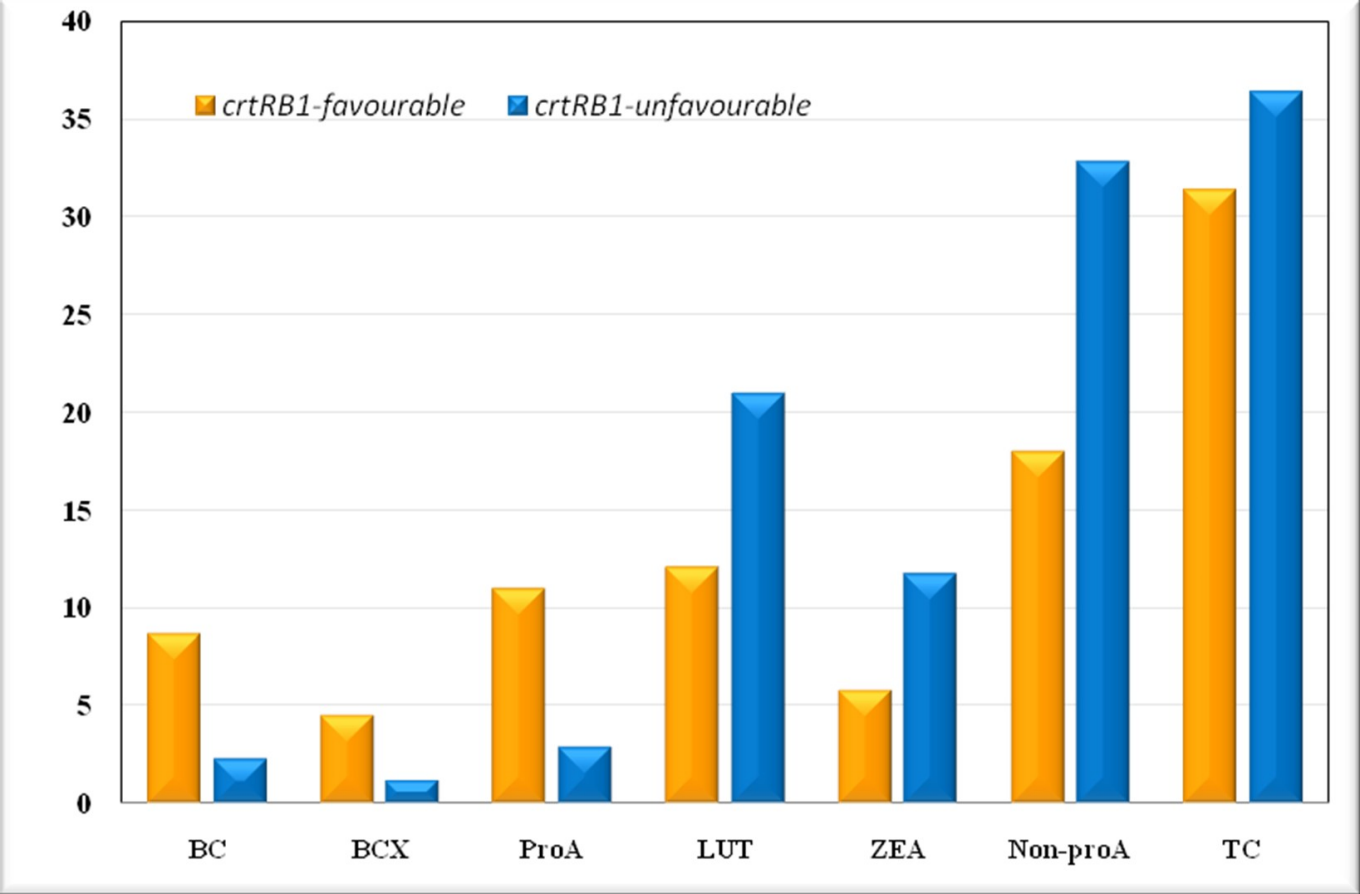
Mean of carotenoids among inbreds with favourable and unfavourable alleles of *crtRB1*.

ANOVA revealed significant variation for BC, BCX, proA, LUT, ZEA, non-proA and total carotenoid (TC) among 24 *crtRB1*-based inbreds and two check inbreds (Table 3). The mean concentration of BC and BCX among the *crtRB1*-based inbreds was 8.72 μg/g and 4.58 μg/g, compared to 2.35 μg/g and 1.24 μg/g in the check inbreds, respectively (Table 4, Fig 4). ProA among the *crtRB1*-based inbred varied from 8.33–14.63 μg/g, with an average of 11.01 μg/g. The elite check inbreds recorded significantly lower levels of proA (mean: 2.97 μg/g). Among 15 inbreds developed under the study, MGU-PVMAS-11 (14.63 μg/g), MGU-PVMAS-5 (13.09 μg/g), MGU-PVMAS-4 (12.53 μg/g), MGU-PVMAS-12 (12.52 μg/g) and MGU-PVMAS-2 (12.28 μg/g) were the most promising ones (Table 4). Six inbreds had proA content of 10–12 μg/g, while rest four inbreds possessed between 8–10 μg/g. The*crtRB1*-based inbreds had significantly low mean LUT (12.16 μg/g), ZEA (5.86 μg/g) and non-proA (18.02 μg/g) compared to check inbreds (LUT: 20.99 μg/g, ZEA: 11.76 μg/g, non-proA: 32.75 μg/g). However,TC was nearly comparable among the *crtRB1*-based (31.31 μg/g) and check inbreds (36.34 μg/g).

**Table 3:** ANOVA of mean sum of squares for carotenoid components among 26 inbred lines.

Source of Variations	df	BC	BCX	ProA	LUT	ZEA	Non-ProA	TC
Replicates	1	0.09 *	0.08	0.18 *	0.12	0.00	0.09	0.07
Genotypes	25	9.00 **	3.33 **	13.87 **	17.70 **	10.33 **	47.39 **	24.49 **
Error	25	0.01	0.06	0.03	0.57	0.03	0.57	0.63
Total	51	4.42	1.66	6.82	8.96	5.08	23.51	12.31

*Significant at p = 0.05

**Significant at p = 0.01, df: Degrees of freedom, BC: β-Carotene, β-cryptoxanthin, ProA: Provitamin-A, LUT: Lutein, Non-ProA: Non-Provitamin-A, TC: Total Carotenoids.

**Table 4:** Mean concentration (μg/g) of carotenoids and grain yield (tha) among inbreds.

S. No.	Inbreds	BC	BCX	ProA	LUT	ZEA	Non-ProA	TC	YLD
1	MGU-PVMAS-1	6.92	2.84	8.33	12.58	4.25	16.82	26.57	3.2
2	MGU-PVMAS-2	9.69	5.18	12.28	13.20	5.60	18.81	33.67	2.6
3	MGU-PVMAS-3	7.66	3.70	9.51	8.50	4.12	12.61	23.97	2.9
4	MGU-PVMAS-4	9.52	6.03	12.53	11.15	4.22	15.37	30.91	3.3
5	MGU-PVMAS-5	10.64	4.90	13.09	11.86	6.87	18.73	34.26	3.3
6	MGU-PVMAS-6	8.20	4.22	10.31	11.13	4.89	16.01	28.44	3.0
7	MGU-PVMAS-7	8.23	4.93	10.70	14.27	8.08	22.34	35.50	3.0
8	MGU-PVMAS-8	7.31	3.56	9.09	11.41	6.24	17.65	28.52	3.1
9	MGU-PVMAS-9	7.69	3.67	9.53	14.99	5.27	20.26	31.62	2.7
10	MGU-PVMAS-10	8.40	4.48	10.64	15.01	4.48	19.48	32.36	3.1
11	MGU-PVMAS-11	12.06	5.14	14.63	9.14	4.51	13.65	30.85	3.4
12	MGU-PVMAS-12	9.82	5.42	12.52	10.49	4.08	14.57	29.80	2.9
13	MGU-PVMAS-13	8.07	4.52	10.33	12.76	7.17	19.93	32.52	3.2
14	MGU-PVMAS-14	7.60	5.39	10.29	14.03	8.18	22.21	35.20	3.5
15	MGU-PVMAS-15	7.96	4.95	10.43	13.36	7.27	20.63	33.53	3.4
16	PMI-PV-1	8.03	4.91	10.48	12.91	4.50	17.41	30.34	2.5
17	PMI-PV-2	9.84	5.88	12.79	10.53	8.25	18.77	34.50	2.3
18	PMI-PV-5	8.06	4.57	10.35	11.42	3.02	14.44	27.07	3.4
19	PMI-PV-6	7.05	5.15	9.63	9.21	3.91	13.12	25.32	3.2
20	PMI-PV-7	8.21	4.20	10.31	12.91	6.57	19.47	31.88	2.9
21	PMI-PV-8	9.84	2.73	11.21	12.94	7.28	20.22	32.79	2.8
22	PMI-PV-9	8.66	2.71	10.02	11.05	8.97	20.01	31.39	3.3
23	HP704-22	10.29	5.67	13.12	13.81	6.73	20.54	36.50	1.4
24	HP465-41	9.53	5.16	12.11	13.17	6.25	19.42	34.10	2.2
Mean		8.72	4.58	11.01	12.16	5.86	18.02	31.31	3.0
Inbreds with unfavourable allele of *crtRB1*									
25	PMI-Q2	2.14	1.30	2.79	19.71	11.24	30.94	34.37	2.9
26	PMI-Q3	2.56	1.18	3.15	22.28	12.29	34.57	38.31	3.3
Mean		2.35	1.24	2.97	20.99	11.76	32.75	36.34	3.0
CD at 5%		0.24	0.50	0.38	1.55	0.35	1.56	1.63	0.6

CD: Critical difference, BC: β-Carotene, β-cryptoxanthin, ProA: Provitamin-A, LUT: Lutein, Non-ProA: Non-Provitamin-A, TC: Total Carotenoids, YLD: Grain yield (t/ha).

Among *crtRB1*-based inbreds, BC and BCX contributed 28% and 15% of TC, while LUT and ZEA contributed 39% and 18%, respectively. The contribution of BC and BCX to TC was only 6% and 3% in check inbreds, while the same for LUT and ZEA was 58% and 33%, respectively.

#### Genotypes × environments interaction for kernel carotenoids

Pooled ANOVA revealed that *crtRB1*-based hybrids had significant variation for BC, BCX, proA, LUT, ZEA, non-proA and TC (Table 5). The proportion of variation contributed by genotypes ranged from 75% (BCX) to 89% (non-proA). Environments though had significant effects on all carotenoids, the magnitude was extremely low (<2% of the total variation). Genotypes **×** environment (G **×** E) interaction was also low with 8% (non-proA) to 22% (BCX).

**Table 5:** Pooled ANOVA for different carotenoids in hybrids across three locations.

Sources of Variation	df	BC	BCX	ProA	LUT	ZEA	Non-ProA	TC
Replicates	1	0.18	0.12	0.06	0.63	0.34	1.88	1.54
Environments	2	9.98 **	4.51 **	15.84 **	9.77**	0.24	8.17 **	45.97 **
Genotypes	79	15.73 **	7.65 **	24.76 **	34.57 **	22.75 **	94.12 **	66.74 **
Genotypes × Environments	158	1.35 **	1.12 **	1.83 **	2.97 **	1.26 **	4.27 **	6.48 **
Error	239	0.19	0.08	0.20	0.57	0.16	0.83	1.19
Total	479	3.17	1.69	4.86	7.01	4.25	17.39	13.93

*Significant at p = 0.05

**Significant at p = 0.01, df: Degrees of Freedom, BC: β- Carotene, BCX: β-Cryptoxanthin, ProA: Provitamin A, LUT: Lutein, ZEA: Zeaxanthin, Non-proA: Non-Provitamin A, TC: Total carotenoid.

#### Variation of carotenoids among experimental hybrids

The mean BC and BCX among experimental hybrids were 8.02 μg/g and 4.69 μg/g, as compared to 2.36 μg/g (BC) and 1.53 μg/g (BCX) among low-proA checks (S4 Table). High-proA checks had 10.06 μg/g (BC) and 3.96 (BCX) μg/g. The concentration for proA among experimental crosses varied from 7.51 to 14.90 μg/g with an average of 10.37 μg/g. The mean concentration among low-proA and high-proA checks was 3.13 and 12.04 μg/g, respectively. Among the experimental hybrids, MGUH-57 with a proA concentration of 14.90 μg/g was the best combination, closely followed by MGUH-1 (14.69 μg/g), MGUH-52 (14.60 μg/g) andMGUH-27 (14.36 μg/g).

The experimental crosses possessed low LUT (mean: 11.22 μg/g, range: 7.41 to 14.46 μg/g) and ZEA (mean: 5.25 μg/g, range: 2.91 to 9.60 μg/g) relative to low-proA checks (LUT: 12.19 μg/g, ZEA: 19.69 μg/g). High-proA checks also possessed low LUT (5.22 μg/g) and ZEA (10.56 μg/g). The non-proA fraction among experimental hybrids varied from 10.78 to 23.50 μg/g, with an average of 16.47 μg/g. The non-proA fraction in low-proA checks was higher (31.88 μg/g) than that of high-proA checks (15.78 μg/g). TC in experimental hybrids varied from 21.92 to 37.86 μg/g, with a mean of 29.18 μg/g. Low-proA checks showed a mean of 35.77 μg/g, while high-proA commercial checks showed a mean of 29.80 μg/g (S4 Table).

**Fig 5:**
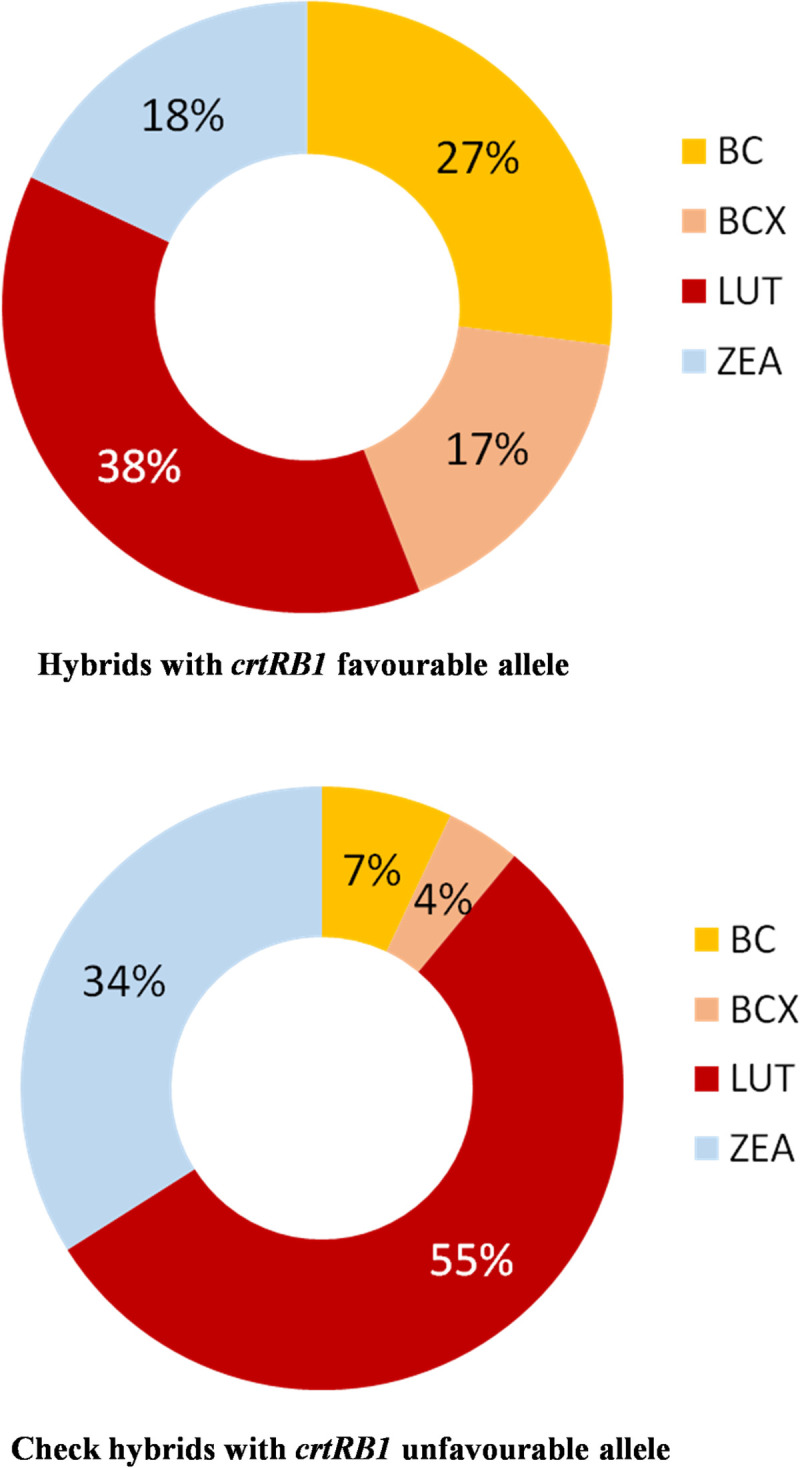
Proportion of carotenoids in hybrids with favourable and unfavourable alleles of *crtRB1*.

In case of experimental hybrids, the contribution of BC and BCX towards TC was 27% and 17%, while LUT and ZEA contributed 38% and 18%, respectively (Fig 5). In case of low-proA checks, the contribution of BC and BCX to TC was only 7% and 4%, respectively. But the contribution was high for LUT (55%) and ZEA (34%). While, in high-proA checks, BC and BCX contributed 34% and 13% to TC, and the contribution of LUT and ZEA was 35% and 18%, respectively.

#### Correlation among carotenoids and grain yield

Correlation analysis revealed that BC was positively correlated with BCX (r = 0.68**). Whereas, BC and BCX were negatively correlated with LUT (r = -0.51, -0.57**) and ZEA (r = -0.42**, -0.53**), respectively. LUT and ZEA, however, were positively correlated (r = 0.70**). ProA and non-proA carotenoids were also negatively correlated (r = -0.57**). BC, BCX and proA were not correlated with TC, while LUT (r = 0.72**), ZEA (r = 0.72**) and non-proA (r = 0.78**) showed strong positive association with TC. The present study revealed that kernel carotenoid components and grain yield exhibited non-significant relationships (r = -0.18 to 0.15) except for ZEA (r = 0.29**).

### Combining ability analysis for carotenoids

#### ANOVA for line × tester

Pooled ANOVA revealed that environment was highly significant for all the carotenoids except for ZEA (Table 6). Line effect was significant only for ZEA, while tester effect was significant for BC, BCX, proA, LUT, non-proA and TC. The interactions of line × tester and environment × crosses were significant for all the carotenoids as well. Environment × line × tester interaction was also found to be significant for all characters except for LUT. On the other hand, environment × tester interaction was significant only for BCX, while environment × line interaction was non-significant for all the characters.

**Table 6:** Pooled ANOVA for carotenoids across three locations in line × tester set.

Source of Variations	df	Mean Sum of Squares						
		BC	BCX	ProA	LUT	ZEA	Non-proA	TC
Replicates	1	0.15	0.09	0.05	1.15	0.38	2.84	2.42
Environments	2	9.25**	5.18**	16.16**	8.48**	0.45	7.70**	46.58**
Rep × Env.	2	1.12**	0.01	1.07**	0.22	0.01	0.27	2.09
Crosses	74	10.57**	6.47**	17.32**	25.04**	16.50**	62.53**	63.95**
Line effect	14	8.60	7.56	16.44	14.93	16.63*	56.52	49.24
Tester effect	4	34.12*	23.47**	53.78*	154.52**	136.13**	497.93**	307.80**
Line × Tester effect	56	9.38**	4.98**	14.94**	18.32**	7.93**	32.94**	50.21**
Env × Crosses	148	1.39**	1.14**	1.90**	3.00**	1.19**	4.33**	6.56**
Env × Line effect	28	1.36	0.88	1.93	2.33	1.63	4.09	8.23
Env × Tester effect	8	1.76	2.90**	2.76	4.36	1.33	7.31	10.90
Env × L × T effect	112	1.36**	1.08**	1.83**	3.07	1.07**	4.17**	5.84**
Error	222	0.17	0.09	0.19	0.59	0.17	0.86	1.19
Total	449	2.33	1.51	3.65	5.45	3.20	12.20	13.51

*Significant at p = 0.05

**Significant at p = 0.01, df: Degrees of Freedom, BC: β- Carotene, BCX: β-Cryptoxanthin, ProA: Provitamin-A, LUT: Lutein, ZEA: Zeaxanthin, Non-proA: Non-Provitamin-A, TC: Total carotenoid.

#### Combining ability estimates

The proportion of additive and dominance variance, and the contribution of lines, testers and line × testers for pooled dataset are presented in Table 7. Variance due to specific combining ability (SCA) was higher than variance due to general combining ability (GCA) for all the characters. Though, dominance variance was predominant for BC, BCX, proA and TC, additive variance was found to be important as well. Additive variance was more for ZEA and non-proA, while both additive and dominance variance was of similar magnitude for LUT. When the contribution of lines, testers and line × tester were compared, line × tester interaction was found to be contributing more than line and testers for all the characters [36.55% (ZEA) to 67.15% (BC)].

**Table 7:** Components of genetic variance and percentage contribution in line × tester set.

Component	BC	BCX	ProA	LUT	ZEA	Non-ProA	TC
σ ^2^ GCA	0.35	0.26	0.58	1.40	1.27	4.61	2.96
σ ^2^ Line × tester (SCA)	1.53	0.82	2.46	2.96	1.29	5.35	8.17
σ ^2^ A (F = 1)	0.71	0.51	1.16	2.80	2.54	9.21	5.91
σ ^2^ D (F = 1)	1.53	0.82	2.46	2.96	1.29	5.35	8.17
σ ^2^ A / σ ^2^ D	0.46	0.63	0.47	0.95	1.97	1.72	0.72
Contribution							
Line	15.40%	22.11%	17.96%	11.28%	19.06%	17.10%	14.57%
Tester	17.45%	19.62%	16.78%	33.36%	44.59%	43.04%	26.02%
Line × Tester	67.15%	58.27%	65.26%	55.37%	36.35%	39.86%	59.41%

ss

#### General combiners for proA

GCA effect for proA varied from -1.52 to 1.27. Nine lines and one tester showed significant positive GCA effects for proA (S5 Table). Among the lines, MGU-PVMAS-10 (1.02) was the best general combiner, followed by MGU-PVMAS-5 (0.64), MGU-PVMAS-1 (0.55) and MGU-PVMAS-11 (0.52) and MGU-PVMAS-15 (0.45). Among testers, PMI-PV-5 (1.27) had the highest GCA effect. Similar observation was also reported for BC and BCX.

### Grain yield among experimental hybrids

Heterosis of the 75 experimental hybrids was estimated over commercial low-proA and high proA checks. CMH08-292 and DHM-121 were medium maturing hybrids with low-proA. ‘Pusa Vivek QPM-9 Improved’ is an early maturing proA rich hybrid and was released as country’s first proA rich maize hybrid during 2017. ‘Pusa HQPM-5 Improved’ and ‘Pusa HQPM-7 Improved’ are the medium maturing proA rich hybrids released in 2020. All the experimental hybrids matured in 92–100 days, thus had medium maturity. The mean grain yield among experimental hybrids was 8.9 t/ha, with the highest yield of 11.4 t/ha (S4 and S5 Tables). Ten experimental hybrids possessed yield between 10 to 11 t/ha, while 25 hybrids had grain yield 9 to 10 t/ha. CoMH08-292 was the best low proA check with 10.4t/ha. A set of 20 experimental hybrids were either better or *at par* with CoMH-08-292. Among high proA checks, ‘Pusa HQPM-5 Improved’ emerged as the best check with 8.6 t/ha. A total of 26 experimental hybrids were significantly better than ‘Pusa HQPM-5 Improved’ for grain yield. These hybrids showed heterosis of 8.04% to 31.63% over the best high proA check.

## Discussion

Plant based-food is the major source of nutrition especially in developing world. Traditional yellow maize lacks the required level of proA [17]. The mutant version of *crtRB1* significantly enhances proA in maize kernel [16]. Diverse proA germplasm has been developed in the tropics [18,19]. However, the genetic base of proA rich inbreds is quite narrow in entire sub-tropics. The frequency of favourable allele of *crtRB1* in the Indian maize germplasm is quite low (3.38%) [32]. So far, 9–10 MABB-derived *crtRB1* inbreds have been developed in India. Thus, targeted breeding approach for selection of *crtRB1* is essential for broadening the genetic base of proA rich maize germplasm [33].

### Marker-assisted selection for *crtRB1*

Genotyping of F_2_ populations indicated that *crtRB1* did not segregate as per the expected 1:2:1. This observation drives strength from earlier results of Lu et al. (2002) [34] and Babu et al. (2013) [17]. The segregation distortion could be due to activity of various gametophytic factors, defective kernel mutants, male sterility and embryo-specific mutation [35]. Since, the frequency of homozygotes for favourable allele was less, raising large backcross populations becomes a necessity for selection of desirable number of positive segregants. Here, *crtRB1* gene could be precisely selected due to the reliable gene-based marker. In case of linked marker, there is always chance of selection of false positive individuals due to crossing over between the gene and marker [36]. The present study developed a set of diverse *crtRB1*-based inbreds using marker-assisted pedigree breeding. Earlier, Muthusamy et al (2014) [37], Liu et al (2015) [38], Zunjare et al (2018) [39] and Goswami et al (2019b) [33] have introgressed *crtRB1* into the elite inbreds using MABB approach. In case of MABB, the improved lines are genetically similar to the recurrent parents except for gene under introgression [36]. In the present study, since *crtRB1*-based inbreds were developed from F_2_ populations, the genetic makeup remains novel leading to development of new and diverse *crtRB1*-based inbreds. The MAS-based selection of *crtRB1* is quite cost-effective, as selection of genotypes for high proA using UHPLC involves US$30–35 per sample. On the contrary, PCR marker-based selection of *crtRB1* employed here costs only US$0.5–1.0 per sample. Molecular breeding is now a preferred choice among the maize breeders to develop proA rich germplasm [2].

### Molecular characterization of *crtRB1*-based inbreds

Knowledge of the genetic relationship among inbreds is essential for efficient exploitation in breeding programme. Molecular markers have been successfully employed to derive the genetic distance in maize [40]. High dissimilarity coefficient indicated the presence of high level of genetic diverseness among the inbreds. Lower major allele frequency also reflects diverse nature of the locus. In the present study, about one-third of total SSR loci had major allele frequency ≤0.5, which indicated large genetic dissimilarity among the inbreds. The genetic information obtained from cluster analysis was highly consistent with pedigree information. PCoA also supported the results of cluster diagram elucidating the diverse nature of inbreds. Inbreds derived from same population source were in general found to be in same cluster [39]. The study also identified few unique alleles and rare alleles among the inbred panel. The identified unique alleles could be useful to distinguish inbreds unambiguously from one another [24,41]. Low mean heterozygosity observed among the SSR loci indicated that inbreds reached appreciable level of homozygosity. Possible reason for heterozygosity observed may be due to tendency of some loci to segregate even after repeated inbreeding [42,43]. Inbreds developed conventionally exhibited higher degree of heterozygosity due to natural selection against homozygotes, when compared with doubled haploid (DH)-based inbreds [44].

### Enrichment of proA due to *crtRB1*

The *crtRB1*-based inbreds and hybrids recorded nearly 3–4 folds more proA (8–14 μg/g) over the traditional checks (2–3 μg/g). Muthusamy et al., (2014) [37] and Zunjare et al., (2018) [39] also reported high proA (8.60–17.50 μg/g and 7.38–13.59 μg/g) among the *crtRB1*-based maize genotypes, respectively. However, traditional yellow maize possesses low concentration of proA (<2.5 ppm) [20,45]. The accumulation of high proA in derived inbreds is due to presence of favourable allele of *crtRB1*, while low proA genotypes harbour the wild-type allele [15,17].

In the carotenoid biosynthesis pathway, *phytoene synthase 1* (*psy1*) or *Yellow 1* (*Y1*) gene condenses two geranyl-geranyl pyrophosphate molecules into one molecule of phytoene [46]. The mutant/recessive *y1* allele is unable to catalyze the reactions and white grains are formed due to no synthesis of carotenoids. However, when the *Y1* is functional, *crtRB1* causes hydroxylation of BC and BCX into ZEA. *CrtRB1* located on chromosome 10 codes for β-carotene hydroxylase. The mutant version of *crtRB1* drastically slows down the conversion, leading to more accumulation of proA carotenoids [16]. Though all the 15 inbreds possessed same *crtRB1* allele, a large variation in proA was observed. This could be due to variation in the activity of other key genes such as *psy1*, *lycopene β-cyclase* (*lcyB*), *lycopene ε-cyclase* (*lcyE*), *phytoene desaturase* (*pds*) and ζ-carotene desaturase (*zds*) catalyzing the carotenoid biosynthesis [37]. Allelic variation for *lcyE* present on chromosome 8 has been observed [47]. Zunjare et al. (2017) [22] reported that the presence of *lcyE* along with *crtRB1* is beneficial in enhancing proA in maize. Besides, several modifier loci/QTLs alone or in combination with other pathway genes could also influence the accumulation of proA in maize [45,48].

### Proportion of carotenoids and their relationships

Among carotenoids, proportion of LUT was higher in both *crtRB1*-based and check- genotypes, thereby, suggesting greater flux of lycopene towards α-branch than the β-branch of pathway [29]. LUT serves as the precursor for various pathways, thus, is required in larger amount. Among check genotypes, ZEA was the second highest carotenoid after LUT. This is due to higher conversion of BC and BCX to ZEA as it serves as the precursor for synthesis of abscisic acid [17]. However, in *crtRB1*-based genotypes, the conversion of BC to ZEA is partially blocked, leading to higher proportion of BC next to LUT. ProA content among the *crtRB1*-based genotypes constituted 43–44% of TC as against 9–11% in the check genotypes. The non-proA component was less (56–57%) among *crtRB1*-based genotypes compared to 89–91% in checks [20,49]. In some of the genotypes, proA with >50% of TC was also observed. This is possibly due to presence of other favourable loci that act synergistically with crtRB1 [29].

BC, BCX and proA showed strong positive correlations, as BCX is produced from BC, while both contribute to proA [49]. Since, BC and BCX are converted to ZEA, negative correlation is expected [50]. Further, LUT in α-branch is produced at the cost of flux of lycopene towards β-branch, where BC and BCX are formed. This mechanism could be responsible for negative relationships among proA components with LUT. ProA carotenoids showed no association with grain yield. This suggested the possibility of developing high yielding maize hybrids with high proA. So far >40 proA rich hybrids and open-pollinated varieties (OPVs) with high grain yield have been developed and commercialized worldwide [2,51].

### Genetics of kernel carotenoids

The current study revealed that environments had minor effect on BC, BCX and proA. Muthusamy et al. (2015b) [41] also reported low effects of environment on carotenoids through analysis of 95 maize lines over the environments. Minor effect of G × E interaction on kernel carotenoids has been reported by Muthusamy et al. (2016) [49] and Goswami et al. (2019a) [29]. The minor effect of environment on kernel carotenoids thus enables identification of potential experimental hybrids adapted over diverse locations [18]. Combining ability is an important area of research in hybrid programmes [52]. It provides useful information in understanding the genetic nature of a trait and aids in selection of suitable parents for superior cross combinations. The result of genetic analysis brought out the importance of both non-additive and additive gene action. In the present study, though dominance variance was predominant for proA, additive variance was important as well. However, earlier studies have reported the predominance of additive gene action on carotenoid accumulation in maize [49,53,54]. This minor variation is possibly due to different germplasm used in the study. This indicated that parental inbreds with high proA may further lead to higher accumulation of proA in hybrids [37]. Various authors have reported that genotype homozygous for favourable allele of *crtRB1* possesses much higher proA compared to heterozygote [16,17,22]. Considering this, both lines and testers were made homozygous for harnessing the benefits of *crtRB1* in all hybrids. Since, all the hybrids were homozygous for favourable allele of *crtRB1*, other modifier loci could be the reason for dominance effects for further influencing the proA. Several lines including testers were identified as the best general combiners for proA. MGUH-57 (14.90 μg/g), MGUH-52 (14.60 μg/g), MGUH-27 (14.36 μg/g) and MGUH-32 (14.26 μg/g) possessed high proA, and had both the parents being high in GCA effects as well. Besides, MGUH-1, MGUH-14, MGUH-15, MGUH-18, MGUH-19, MGUH-28, MGUH-31, MGUH-48, MGUH-50 and MGUH-75 had one of the parents (used as line) having high GCA effects for proA. The inbreds with high GCA for proA thus serve as a promising inbreds in the future breeding programme [49].

### Promising high yielding proA rich hybrids

Several promising experimental hybrids with >10.0 t/ha grain yield and >10.0 μg/g proA were identified. MGUH-15 (grain yield: 11.4t/ha, proA: 11.32 μg/g), MGUH-50 (grain yield: 10.3t/ha, proA: 11.44 μg/g), MGUH-72 (grain yield: 10.1 t/ha, proA: 11.87 μg/g), MGUH-54 (grain yield: 10.2t/ha, proA: 9.25 μg/g) and MGUH-55 (grain yield: 10.3t/ha, proA: 10.10 μg/g) were the most promising combinations. Most of these promising hybrids belongs to different clusters. These hybrids are much higher yielding than the ‘Pusa Vivek QPM-9 Improved’ (grain yield: 7.2t/ha, proA: 11.83 μg/g), the first proA rich hybrid released in India. This is primarily due to extra-early maturity of ‘Pusa Vivek QPM-9 Improved’. Besides, two of the proA hybrids, ‘Pusa HQPM5 Improved’ (grain yield: 8.6t/ha, proA: 11.85 μg/g) and ‘Pusa HQPM7 Improved’ (grain yield: 8.5t/ha, proA: 12.45 μg/g) in the medium maturity groups have been recently released. Thus, these identified hybrids are higher yielding than the proA check hybrids as well. These selected hybrids also possessed higher proA than the normal best check (CoMH08-292 and DHM-121), but were *at par* with CoMH08-292 (grain yield: 10.4t/ha, proA: 3.27 μg/g) and significantly better than DHM-121 (grain yield: 8.6t/ha, 2.99 μg/g) for grain yield potential.

Further, parents of these proA rich hybrids also produce high yield from seed production perspective. The average grain yield among parental inbreds (used as lines) developed under this programme was 3.1t/ha with a range of 2.6–3.5t/ha. The same in *crtRB1*-donor was only 1.4t/ha, thereby suggesting its poor adaptability in subtropical conditions. The high grain yield of new *crtRB1*-based inbreds depicts better adaptability. The high yielding proA rich hybrids identified here would thus provide more productivity and profit to the farmers, and offer higher vitamin-A to the consumers [2]. Further, chickens accumulate more proA in egg yolk when fed with proA rich biofortified maize grains [55]. The proA rich maize used directly as food and indirectly through eggs would provide sufficient vitamin-A required for proper growth and development in humans. These proA rich hybrids thus, assume great significance in food and nutritional security, and would play important role in alleviating VAD in the country.

## Conclusion

Diverse *crtRB1*-based inbreds have been developed in the study through marker-assisted pedigree breeding. These new inbreds possessed significantly higher proA than the checks. Molecular characterization of the inbreds depicted their diverse genetic nature. Genetic analysis revealed that both additive and non-additive variances were important. The *crtRB1*-based inbreds were successfully used in development of proA rich hybrids. Promising high yielding hybrids with very high concentration of proA have been identified. These proA rich hybrids are higher yielding than the exiting proA checks and *at par* with the normal checks in grain yield but with higher proA. The present study demonstrated the successful application of markers-assisted pedigree breeding in broadening the genetic base, and developing promising hybrids with higher grain yield as well as improved nutritional quality.

## Supporting information

S1 Table List of diverse maize inbreds used for studying effect of *crtRB1*. Q: Represents the *opaque2* versions, TNAU: Tamil Nadu Agricultural University; ANGRAU: Acharya N. G. Ranga Agricultural University; PAU: Punjab Agricultural University.
(DOC)

S2 Table Details of inbred lines used for carotenoid estimation and molecular characterization. (DOCX)

S3 Table Jaccard’s dissimilarity coefficient among 24 proA maize inbreds using based on SSR dataset. 1. MGU-PVMAS-13, 2. PMI-PV-8, 3. PMI-PV-7, 4. MGU-PVMAS-6, 5. MGU-PVMAS-5, 6. MGU-PVMAS-11, 7. MGU-PVMAS-9, 8. MGU-PVMAS-4, 9. MGU-PVMAS-8, 10. MGU-PVMAS-15, 11. MGU-PVMAS-14, 12. MGU-PVMAS-7, 13. HP465-41, 14. PMI-PV-1, 15. MGU-PVMAS-3, 16. MGU-PVMAS-2, 17. MGU-PVMAS-12, 18. MGU-PVMAS-10, 19. HP704-22, 20. PMI-PV-6, 21. PMI-PV-9, 22. PMI-PV-5, 23. PMI-PV-2, 24. MGU-PVMAS-1, Inb. = Inbred.
(DOCX)

S4 Table Mean of different carotenoids (μg/g) and grain yield (t/ha) of 80 hybrids across three locations. BC: β- Carotene, BCX: β-Cryptoxanthin, ProA: Provitamin A, LUT: Lutein, ZEA: Zeaxanthin, Non-ProA: Non-Provitamin A, TC: Total carotenoids, YLD: Yield.
(DOCX)

S5 Table GCA effects for different carotenoids of 20 parental inbreds across three locations. *Significant at p = 0.05; **Significant at p = 0.01, BC: β- Carotene, BCX: β-Cryptoxanthin, ProA: Provitamin-A, LUT: Lutein, ZEA: Zeaxanthin, Non-ProA: Non-Provitamin-A, TC: Total carotenoids, SE: Standard Error.
(DOCX)

S6 Table Percent economic heterosis of experimental hybrids over commercial checks across the locations. *Significant at p = 0.05; **Significant at p = 0.01.
(DOCX)

S1 File Segregation of favourable (543 bp) and unfavourable (296 bp) alleles of *crtRB1* in F_2_ populations. (DOCX)
