# Supplementary material for: Development of sub-tropically adapted diverse provitamin-A rich maize inbreds through marker-assisted pedigree selection, their characterization and utilization in hybrid breeding
Source: PLoS One. 2021 Feb 4;16(2):e0245497. doi: 10.1371/journal.pone.0245497 (PMC7861415; doi:10.1371/journal.pone.0245497)
Supplement: S1 Table — Q: Represents the opaque2 versions, TNAU: Tamil Nadu Agricultural University; ANGRAU: Acharya N. G. Ranga Agricultural University; PAU: Punjab Agricultural University. (DOC) [file pone.0245497.s001.doc]

**Table S1.** **List of diverse maize inbreds used for studying effect of *crtRB1***.

| **S. No.** | **Genotypes** | **Source population** | **Source Institution** |
| --- | --- | --- | --- |
| Recipient parents | | | |
| 1. | UMI-1200 | Selection from W2619-3 | TNAU, Coimbatore |
| 2. | UMI-1230 | Selection from C7254-105-3 | TNAU, Coimbatore |
| 3. | BML-6Q | SRRL65-B96 | ANGRAU, Hyderabad |
| 4. | BML-7Q | {X2Y Pool × CML226}-B98 R | ANGRAU, Hyderabad |
| 5. | LM-11Q | SW1-26 | PAU, Ludhiana |
| 6. | LM-12Q | J54 Mo17 | PAU, Ludhiana |
| 7. | LM-13Q | LCY3 | PAU, Ludhiana |
| 8. | LM-14Q | CA00310 | PAU, Ludhiana |
| 9. | PDM-4341 | (Comp8551 X Comp 8527xAgeti76X MDR) -9- 4-2-8-7-1-1-2-1-R-3 | RRS-IARI, Dharwad |
| 10. | PDM-4251 | PS-25-1-1-1-1-1-1-1-1-R | RRS-IARI, Dharwad |
| Donor parent | | | |
| 11. | HP704-22 | (KUI carotenoid syn-FS11-1-1-B-B-B/(KU1409/DE3/KU1409)S2-18-2-B)-B-3(MAS:L4H1)-1-B-B-B | CIMMYT HarvestPlus |

Q: Represents the *opaque2* versions

TNAU: Tamil Nadu Agricultural University; ANGRAU: Acharya N. G. Ranga Agricultural University; PAU: Punjab Agricultural University
