# Supplementary material for: Development of sub-tropically adapted diverse provitamin-A rich maize inbreds through marker-assisted pedigree selection, their characterization and utilization in hybrid breeding
Source: PLoS One. 2021 Feb 4;16(2):e0245497. doi: 10.1371/journal.pone.0245497 (PMC7861415; doi:10.1371/journal.pone.0245497)
Supplement: S2 Table — (DOCX) [file pone.0245497.s002.docx]

**Table S2. Details of inbred lines used for carotenoid estimation and molecular characterization.**

| **S. No.** | **Genotypes** | **Pedigree** | **Source Institution** |
| --- | --- | --- | --- |
| Inbreds with favorable allele of *crtRB1* | | | |
| 1 | MGU-PVMAS-1 | (UMI1200/HP704-22)-54-3-⊗-⊗ | IARI, New Delhi |
| 2 | MGU-PVMAS-2 | (UMI1200/HP704-22)-56-1-⊗-⊗ | IARI, New Delhi |
| 3 | MGU-PVMAS-3 | (UMI1230/HP704-22)-4-3-⊗-⊗ | IARI, New Delhi |
| 4 | MGU-PVMAS-4 | (UMI1230/HP704-22)-46-5-⊗-⊗ | IARI, New Delhi |
| 5 | MGU-PVMAS-5 | (BML6-Q/HP704-22)-19-3-⊗-⊗ | IARI, New Delhi |
| 6 | MGU-PVMAS-6 | (BML6-Q/HP704-22)-19-5-⊗-⊗ | IARI, New Delhi |
| 7 | MGU-PVMAS-7 | (BML7-Q/HP704-22)-32-1-⊗-⊗ | IARI, New Delhi |
| 8 | MGU-PVMAS-8 | (BML7-Q/HP704-22)-80-2-⊗-⊗ | IARI, New Delhi |
| 9 | MGU-PVMAS-9 | (LM11-Q/HP704-22)-2-3-⊗-⊗ | IARI, New Delhi |
| 10 | MGU-PVMAS-10 | (HP704-22/LM12-Q)-24-5-⊗-⊗ | IARI, New Delhi |
| 11 | MGU-PVMAS-11 | (LM13-Q/HP704-22)-14-5-⊗-⊗ | IARI, New Delhi |
| 12 | MGU-PVMAS-12 | (LM13-Q/HP704-22)-51-5-⊗-⊗ | IARI, New Delhi |
| 13 | MGU-PVMAS-13 | (LM14-Q/HP704-22)-20-2-⊗-⊗ | IARI, New Delhi |
| 14 | MGU-PVMAS-14 | (AH7000F/HP704-22)-56-5-⊗-⊗ | IARI, New Delhi |
| 15 | MGU-PVMAS-15 | (AH7000M/HP704-22)-10-1-⊗-⊗ | IARI, New Delhi |
| 16 | PMI-PV-1 | (VQL1///HP465-43)-⊗-⊗-⊗-⊗-⊗-⊗-⊗-⊗ | IARI, New Delhi |
| 17 | PMI-PV-2 | (VQL2//HP465-41) ⊗-⊗-⊗-⊗-⊗-⊗-⊗-⊗ | IARI, New Delhi |
| 18 | PMI-PV-5 | (HKI161/// HP704-23)-⊗-⊗-⊗-⊗-⊗-⊗ | IARI, New Delhi |
| 19 | PMI-PV-6 | (HKI163///HP704-22)-⊗-⊗-⊗-⊗-⊗-⊗ | IARI, New Delhi |
| 20 | PMI-PV-7 | (HKI193-1///HP704-23)-⊗-⊗-⊗-⊗-⊗-⊗ | IARI, New Delhi |
| 21 | PMI-PV-8 | (HKI193-2///HP704-22)-⊗-⊗-⊗-⊗-⊗-⊗ | IARI, New Delhi |
| 22 | PMI-PV-9 | (HKI1105Q/HKI1105PV)-⊗-⊗-⊗-⊗-⊗-⊗ | IARI, New Delhi |
| 23 | HP704-22 | (KUI carotenoid syn-FS11-1-1-B-B-B/(KU1409/DE3/KU1409) S2-18-2-B)-B-3(MAS: L4H1)-1-B-B-B | CIMMYT, Mexico |
| 24 | HP465-41 | (KUI carotenoid syn-FS25-3-2-B-B-B/(KU1409/DE3/KU1409) S2-18-2-B)-B-2(MAS: L4H1)-1 | CIMMYT, Mexico |
| Inbreds with unfavourable allele of *crtRB1* | | | |
| 25 | PMI-Q2 | HKI323Q | IARI, New Delhi |
| 26 | PMI-Q3 | HKI1128Q | IARI, New Delhi |
