# Supplementary material for: Development of sub-tropically adapted diverse provitamin-A rich maize inbreds through marker-assisted pedigree selection, their characterization and utilization in hybrid breeding
Source: PLoS One. 2021 Feb 4;16(2):e0245497. doi: 10.1371/journal.pone.0245497 (PMC7861415; doi:10.1371/journal.pone.0245497)
Supplement: S3 Table — 1. MGU-PVMAS-13, 2. PMI-PV-8, 3. PMI-PV-7, 4. MGU-PVMAS-6, 5. MGU-PVMAS-5, 6. MGU-PVMAS-11, 7. MGU-PVMAS-9, 8. MGU-PVMAS-4, 9. MGU-PVMAS-8, 10. MGU-PVMAS-15, 11. MGU-PVMAS-14, 12. MGU-PVMAS-7, 13. HP465-41, 14. PMI-PV-1, 15. MGU-PVMAS-3, 16. MGU-PVMAS-2, 17. MGU-PVMAS-12, 18. MGU-PVMAS-10, 19. HP704-22, 20. PMI-PV-6, 21. PMI-PV-9, 22. PMI-PV-5, 23. PMI-PV-2, 24. MGU-PVMAS-1, Inb. = Inbred. (DOCX) [file pone.0245497.s003.docx]

**Table S3.** **Jaccard’s dissimilarity coefficient among 24 proA maize inbreds using based on SSR dataset.**

| **Inb.** | **1** | **2** | **3** | **4** | **5** | **6** | **7** | **8** | **9** | **10** | **11** | **12** | **13** | **14** | **15** | **16** | **17** | **18** | **19** | **20** | **21** | **22** | **23** |
| --- | --- | --- | --- | --- | --- | --- | --- | --- | --- | --- | --- | --- | --- | --- | --- | --- | --- | --- | --- | --- | --- | --- | --- |
| **2** | 0.72 |  |  |  |  |  |  |  |  |  |  |  |  |  |  |  |  |  |  |  |  |  |  |
| **3** | 0.71 | 0.32 |  |  |  |  |  |  |  |  |  |  |  |  |  |  |  |  |  |  |  |  |  |
| **4** | 0.70 | 0.62 | 0.64 |  |  |  |  |  |  |  |  |  |  |  |  |  |  |  |  |  |  |  |  |
| **5** | 0.68 | 0.63 | 0.65 | 0.28 |  |  |  |  |  |  |  |  |  |  |  |  |  |  |  |  |  |  |  |
| **6** | 0.70 | 0.66 | 0.68 | 0.70 | 0.68 |  |  |  |  |  |  |  |  |  |  |  |  |  |  |  |  |  |  |
| **7** | 0.66 | 0.63 | 0.64 | 0.59 | 0.59 | 0.61 |  |  |  |  |  |  |  |  |  |  |  |  |  |  |  |  |  |
| **8** | 0.71 | 0.70 | 0.67 | 0.68 | 0.64 | 0.68 | 0.60 |  |  |  |  |  |  |  |  |  |  |  |  |  |  |  |  |
| **9** | 0.84 | 0.71 | 0.71 | 0.66 | 0.66 | 0.71 | 0.67 | 0.65 |  |  |  |  |  |  |  |  |  |  |  |  |  |  |  |
| **10** | 0.77 | 0.73 | 0.72 | 0.64 | 0.67 | 0.65 | 0.55 | 0.61 | 0.61 |  |  |  |  |  |  |  |  |  |  |  |  |  |  |
| **11** | 0.72 | 0.72 | 0.69 | 0.64 | 0.66 | 0.66 | 0.62 | 0.69 | 0.71 | 0.58 |  |  |  |  |  |  |  |  |  |  |  |  |  |
| **12** | 0.77 | 0.72 | 0.72 | 0.64 | 0.66 | 0.73 | 0.69 | 0.69 | 0.57 | 0.75 | 0.64 |  |  |  |  |  |  |  |  |  |  |  |  |
| **13** | 0.62 | 0.70 | 0.66 | 0.60 | 0.64 | 0.65 | 0.57 | 0.64 | 0.64 | 0.51 | 0.63 | 0.66 |  |  |  |  |  |  |  |  |  |  |  |
| **14** | 0.73 | 0.65 | 0.69 | 0.66 | 0.63 | 0.74 | 0.65 | 0.80 | 0.71 | 0.72 | 0.73 | 0.75 | 0.71 |  |  |  |  |  |  |  |  |  |  |
| **15** | 0.60 | 0.70 | 0.67 | 0.59 | 0.62 | 0.71 | 0.61 | 0.59 | 0.72 | 0.66 | 0.68 | 0.73 | 0.61 | 0.70 |  |  |  |  |  |  |  |  |  |
| **16** | 0.80 | 0.68 | 0.67 | 0.64 | 0.58 | 0.61 | 0.61 | 0.60 | 0.58 | 0.60 | 0.61 | 0.67 | 0.59 | 0.73 | 0.61 |  |  |  |  |  |  |  |  |
| **17** | 0.74 | 0.73 | 0.73 | 0.69 | 0.68 | 0.66 | 0.66 | 0.75 | 0.78 | 0.71 | 0.72 | 0.75 | 0.68 | 0.74 | 0.70 | 0.58 |  |  |  |  |  |  |  |
| **18** | 0.73 | 0.69 | 0.70 | 0.55 | 0.56 | 0.61 | 0.58 | 0.60 | 0.71 | 0.61 | 0.66 | 0.68 | 0.56 | 0.70 | 0.59 | 0.55 | 0.55 |  |  |  |  |  |  |
| **19** | 0.70 | 0.69 | 0.70 | 0.49 | 0.56 | 0.63 | 0.55 | 0.62 | 0.69 | 0.61 | 0.61 | 0.63 | 0.50 | 0.69 | 0.55 | 0.52 | 0.57 | 0.42 |  |  |  |  |  |
| **20** | 0.64 | 0.70 | 0.72 | 0.69 | 0.69 | 0.73 | 0.69 | 0.72 | 0.70 | 0.70 | 0.67 | 0.68 | 0.70 | 0.69 | 0.65 | 0.71 | 0.71 | 0.68 | 0.59 |  |  |  |  |
| **21** | 0.72 | 0.72 | 0.70 | 0.71 | 0.65 | 0.68 | 0.70 | 0.74 | 0.78 | 0.76 | 0.73 | 0.75 | 0.66 | 0.70 | 0.71 | 0.68 | 0.65 | 0.70 | 0.61 | 0.63 |  |  |  |
| **22** | 0.76 | 0.71 | 0.73 | 0.73 | 0.76 | 0.76 | 0.77 | 0.74 | 0.72 | 0.77 | 0.82 | 0.76 | 0.69 | 0.78 | 0.68 | 0.75 | 0.77 | 0.71 | 0.69 | 0.56 | 0.70 |  |  |
| **23** | 0.76 | 0.75 | 0.74 | 0.67 | 0.70 | 0.80 | 0.69 | 0.67 | 0.77 | 0.73 | 0.78 | 0.79 | 0.73 | 0.77 | 0.74 | 0.75 | 0.80 | 0.74 | 0.68 | 0.67 | 0.76 | 0.68 |  |
| **24** | 0.69 | 0.71 | 0.70 | 0.64 | 0.68 | 0.74 | 0.67 | 0.71 | 0.74 | 0.71 | 0.64 | 0.74 | 0.65 | 0.71 | 0.68 | 0.63 | 0.70 | 0.73 | 0.67 | 0.70 | 0.74 | 0.74 | 0.65 |

1.MGU-PVMAS-13, 2. PMI-PV-8, 3. PMI-PV-7, 4. MGU-PVMAS-6, 5. MGU-PVMAS-5, 6. MGU-PVMAS-11, 7. MGU-PVMAS-9, 8. MGU-PVMAS-4, 9. MGU-PVMAS-8, 10. MGU-PVMAS-15, 11. MGU-PVMAS-14, 12. MGU-PVMAS-7, 13. HP465-41, 14. PMI-PV-1, 15. MGU-PVMAS-3, 16. MGU-PVMAS-2, 17. MGU-PVMAS-12, 18. MGU-PVMAS-10, 19. HP704-22, 20. PMI-PV-6, 21. PMI-PV-9, 22. PMI-PV-5, 23. PMI-PV-2, 24. MGU-PVMAS-1, Inb.=Inbred
