# Supplementary material for: Development of sub-tropically adapted diverse provitamin-A rich maize inbreds through marker-assisted pedigree selection, their characterization and utilization in hybrid breeding
Source: PLoS One. 2021 Feb 4;16(2):e0245497. doi: 10.1371/journal.pone.0245497 (PMC7861415; doi:10.1371/journal.pone.0245497)
Supplement: S4 Table — BC: β- Carotene, BCX: β-Cryptoxanthin, ProA: Provitamin A, LUT: Lutein, ZEA: Zeaxanthin, Non-ProA: Non-Provitamin A, TC: Total carotenoids, YLD: Yield. (DOCX) [file pone.0245497.s004.docx]

**Table S4.** **Mean of different carotenoids (µg/g) and grain yield (t/ha) of 80 hybrids across three locations.**

| **S. No.** | **LxT code** | **Crosses** | **BC** | **BCX** | **ProA** | **LUT** | **ZEA** | **Non-proA** | **TC** | **YLD** |
| --- | --- | --- | --- | --- | --- | --- | --- | --- | --- | --- |
| 1 | L1 × T1 | MGUH-1 | 10.95 | 7.48 | 14.69 | 8.59 | 4.06 | 12.65 | 31.07 | 8.9 |
| 2 | L1 × T2 | MGUH-2 | 8.04 | 6.28 | 11.18 | 13.41 | 4.35 | 17.76 | 32.08 | 9.7 |
| 3 | L1 × T3 | MGUH-3 | 6.52 | 4.62 | 8.83 | 7.86 | 2.91 | 10.78 | 21.92 | 8.8 |
| 4 | L1 × T4 | MGUH-4 | 8.71 | 4.17 | 10.79 | 7.66 | 3.20 | 10.86 | 22.63 | 8.3 |
| 5 | L1 × T5 | MGUH-5 | 8.23 | 2.63 | 9.55 | 13.50 | 5.36 | 18.86 | 29.73 | 9.1 |
| 6 | L2 × T1 | MGUH-6 | 6.01 | 3.63 | 7.83 | 12.41 | 5.16 | 17.56 | 27.21 | 7.7 |
| 7 | L2 × T2 | MGUH-7 | 8.02 | 4.31 | 10.18 | 11.96 | 3.70 | 15.65 | 27.99 | 8.4 |
| 8 | L2 × T3 | MGUH-8 | 6.40 | 3.73 | 8.27 | 9.27 | 4.45 | 13.72 | 23.85 | 8.4 |
| 9 | L2 × T4 | MGUH-9 | 6.32 | 3.98 | 8.31 | 9.46 | 3.77 | 13.22 | 23.53 | 9.5 |
| 10 | L2 × T5 | MGUH-10 | 7.77 | 3.81 | 9.67 | 11.32 | 6.23 | 17.55 | 29.13 | 10.4 |
| 11 | L3 × T1 | MGUH-11 | 5.57 | 3.88 | 7.51 | 13.06 | 4.22 | 17.28 | 26.73 | 8.7 |
| 12 | L3 × T2 | MGUH-12 | 8.32 | 4.70 | 10.67 | 7.53 | 5.21 | 12.74 | 25.76 | 8.4 |
| 13 | L3 × T3 | MGUH-13 | 7.49 | 5.42 | 10.20 | 10.37 | 4.43 | 14.80 | 27.71 | 9.2 |
| 14 | L3 × T4 | MGUH-14 | 10.30 | 6.62 | 13.61 | 9.12 | 3.41 | 12.54 | 29.45 | 7.6 |
| 15 | L3 × T5 | MGUH-15 | 8.78 | 5.08 | 11.32 | 12.71 | 7.29 | 20.00 | 32.31 | 11.4 |
| 16 | L4 × T1 | MGUH-16 | 8.11 | 4.15 | 10.19 | 11.21 | 4.58 | 15.79 | 28.05 | 8.6 |
| 17 | L4 × T2 | MGUH-17 | 7.67 | 4.53 | 9.93 | 10.75 | 4.86 | 15.61 | 27.81 | 9.8 |
| 18 | L4 × T3 | MGUH-18 | 10.05 | 6.10 | 13.10 | 13.51 | 6.44 | 19.95 | 36.10 | 9.4 |
| 19 | L4 × T4 | MGUH-19 | 9.17 | 5.98 | 12.16 | 7.41 | 3.60 | 11.00 | 26.15 | 10.0 |
| 20 | L4 × T5 | MGUH-20 | 6.61 | 3.16 | 8.19 | 14.08 | 9.35 | 23.43 | 33.20 | 9.5 |
| 21 | L5 × T1 | MGUH-21 | 7.65 | 3.50 | 9.39 | 13.56 | 4.62 | 18.18 | 29.32 | 9.1 |
| 22 | L5 × T2 | MGUH-22 | 8.46 | 3.95 | 10.44 | 9.13 | 4.96 | 14.09 | 25.18 | 10.0 |
| 23 | L5 × T3 | MGUH-23 | 7.92 | 5.39 | 10.62 | 9.22 | 4.14 | 13.36 | 26.67 | 10.0 |
| 24 | L5 × T4 | MGUH-24 | 7.20 | 5.17 | 9.79 | 10.52 | 4.35 | 14.87 | 27.24 | 9.8 |
| 25 | L5 × T5 | MGUH-25 | 7.62 | 3.90 | 9.57 | 13.64 | 5.71 | 19.35 | 30.87 | 10.0 |
| 26 | L6 × T1 | MGUH-26 | 7.79 | 3.91 | 9.74 | 9.33 | 4.50 | 13.84 | 25.54 | 7.4 |
| 27 | L6 × T2 | MGUH-27 | 11.56 | 5.61 | 14.36 | 12.89 | 4.49 | 17.38 | 34.55 | 7.2 |
| 28 | L6 × T3 | MGUH-28 | 8.76 | 6.10 | 11.81 | 8.49 | 5.07 | 13.57 | 28.42 | 8.8 |
| 29 | L6 × T4 | MGUH-29 | 7.72 | 5.76 | 10.60 | 10.31 | 3.84 | 14.15 | 27.63 | 8.3 |
| 30 | L6 × T5 | MGUH-30 | 8.42 | 4.00 | 10.42 | 13.81 | 8.37 | 22.18 | 33.72 | 9.4 |
| 31 | L7 × T1 | MGUH-31 | 8.82 | 4.63 | 11.13 | 12.89 | 4.34 | 17.23 | 30.68 | 8.5 |
| 32 | L7 × T2 | MGUH-32 | 10.89 | 6.73 | 14.26 | 10.31 | 5.21 | 15.52 | 33.15 | 7.9 |
| 33 | L7 × T3 | MGUH-33 | 6.69 | 4.74 | 9.06 | 11.61 | 4.86 | 16.46 | 27.89 | 8.2 |
| 34 | L7 × T4 | MGUH-34 | 7.48 | 4.69 | 9.83 | 11.44 | 4.47 | 15.91 | 28.08 | 8.4 |
| 35 | L7 × T5 | MGUH-35 | 7.94 | 4.45 | 10.16 | 11.47 | 6.92 | 18.40 | 30.78 | 9.0 |
| 36 | L8 × T1 | MGUH-36 | 6.47 | 4.09 | 8.52 | 12.94 | 6.31 | 19.25 | 29.81 | 8.0 |
| 37 | L8 × T2 | MGUH-37 | 9.26 | 3.50 | 11.01 | 9.97 | 4.63 | 14.60 | 26.60 | 9.0 |
| 38 | L8 × T3 | MGUH-38 | 6.13 | 4.67 | 8.47 | 9.13 | 5.66 | 14.78 | 25.58 | 8.4 |
| 39 | L8 × T4 | MGUH-39 | 5.88 | 4.73 | 8.24 | 13.56 | 4.68 | 18.24 | 28.84 | 8.7 |
| 40 | L8 × T5 | MGUH-40 | 7.19 | 4.36 | 9.37 | 14.21 | 8.57 | 22.78 | 34.33 | 8.7 |
| 41 | L9 × T1 | MGUH-41 | 7.64 | 4.10 | 9.70 | 13.10 | 3.51 | 16.61 | 28.36 | 6.9 |
| 42 | L9 × T2 | MGUH-42 | 8.68 | 5.89 | 11.62 | 8.92 | 3.68 | 12.60 | 27.16 | 9.7 |
| 43 | L9 × T3 | MGUH-43 | 7.47 | 3.64 | 9.29 | 11.89 | 5.10 | 16.98 | 28.09 | 7.9 |
| 44 | L9 × T4 | MGUH-44 | 9.11 | 5.84 | 12.03 | 11.16 | 4.59 | 15.75 | 30.70 | 9.3 |
| 45 | L9 × T5 | MGUH-45 | 7.50 | 4.25 | 9.62 | 14.30 | 7.68 | 21.98 | 33.72 | 9.2 |
| 46 | L10 × T1 | MGUH-46 | 7.45 | 3.26 | 9.08 | 13.68 | 4.31 | 17.99 | 28.70 | 8.0 |
| 47 | L10 × T2 | MGUH-47 | 9.30 | 5.37 | 11.98 | 11.59 | 4.49 | 16.08 | 30.75 | 5.5 |
| 48 | L10 × T3 | MGUH-48 | 8.45 | 5.91 | 11.41 | 14.30 | 9.20 | 23.50 | 37.86 | 8.5 |
| 49 | L10 × T4 | MGUH-49 | 6.97 | 5.13 | 9.53 | 8.42 | 3.61 | 12.03 | 24.13 | 10.0 |
| 50 | L10 × T5 | MGUH-50 | 9.42 | 4.05 | 11.44 | 14.46 | 8.99 | 23.44 | 34.70 | 10.3 |
| 51 | L11 × T1 | MGUH-51 | 6.23 | 3.46 | 7.96 | 14.01 | 5.26 | 19.26 | 28.95 | 8.0 |
| 52 | L11 × T2 | MGUH-52 | 11.27 | 6.67 | 14.60 | 9.06 | 4.33 | 13.39 | 31.32 | 7.9 |
| 53 | L11 × T3 | MGUH-53 | 9.37 | 5.66 | 12.20 | 11.71 | 3.44 | 15.16 | 30.18 | 9.0 |
| 54 | L11 × T4 | MGUH-54 | 7.06 | 4.38 | 9.25 | 9.07 | 4.86 | 13.93 | 25.37 | 10.2 |
| 55 | L11 × T5 | MGUH-55 | 8.28 | 3.64 | 10.10 | 10.71 | 9.14 | 19.84 | 31.76 | 10.3 |
| 56 | L12 × T1 | MGUH-56 | 6.50 | 4.55 | 8.77 | 13.76 | 5.35 | 19.12 | 30.16 | 8.2 |
| 57 | L12 × T2 | MGUH-57 | 11.20 | 7.40 | 14.90 | 9.43 | 4.27 | 13.70 | 32.31 | 8.1 |
| 58 | L12 × T3 | MGUH-58 | 7.53 | 4.98 | 10.01 | 8.97 | 3.37 | 12.34 | 24.85 | 9.0 |
| 59 | L12 × T4 | MGUH-59 | 7.67 | 5.78 | 10.55 | 10.55 | 3.63 | 14.18 | 27.62 | 9.3 |
| 60 | L12 × T5 | MGUH-60 | 8.00 | 4.67 | 10.33 | 8.73 | 7.63 | 16.35 | 29.02 | 9.6 |
| 61 | L13 × T1 | MGUH-61 | 7.58 | 3.30 | 9.23 | 14.09 | 5.81 | 19.90 | 30.78 | 7.7 |
| 62 | L13 × T2 | MGUH-62 | 7.30 | 3.58 | 9.09 | 9.58 | 4.84 | 14.42 | 25.30 | 9.3 |
| 63 | L13 × T3 | MGUH-63 | 8.75 | 3.88 | 10.69 | 12.37 | 9.60 | 21.97 | 33.56 | 9.5 |
| 64 | L13 × T4 | MGUH-64 | 6.77 | 4.13 | 8.84 | 11.62 | 5.70 | 17.31 | 28.21 | 9.9 |
| 65 | L13 × T5 | MGUH-65 | 7.63 | 3.36 | 9.31 | 13.78 | 9.44 | 23.22 | 34.21 | 10.0 |
| 66 | L14 × T1 | MGUH-66 | 8.50 | 3.50 | 10.25 | 14.33 | 6.24 | 20.57 | 32.56 | 9.0 |
| 67 | L14 × T2 | MGUH-67 | 6.63 | 3.82 | 8.54 | 10.18 | 4.69 | 14.87 | 25.33 | 8.7 |
| 68 | L14 × T3 | MGUH-68 | 8.49 | 4.94 | 10.96 | 9.57 | 5.35 | 14.92 | 28.34 | 8.9 |
| 69 | L14 × T4 | MGUH-69 | 7.75 | 4.27 | 9.88 | 9.09 | 5.03 | 14.13 | 26.14 | 10.5 |
| 70 | L14 × T5 | MGUH-70 | 7.75 | 4.97 | 10.24 | 12.65 | 4.57 | 17.22 | 29.94 | 7.6 |
| 71 | L15 × T1 | MGUH-71 | 6.81 | 4.58 | 9.10 | 12.22 | 6.14 | 18.36 | 29.75 | 8.2 |
| 72 | L15 × T2 | MGUH-72 | 9.10 | 5.54 | 11.87 | 9.20 | 4.65 | 13.84 | 28.48 | 10.0 |
| 73 | L15 × T3 | MGUH-73 | 7.34 | 4.85 | 9.76 | 10.34 | 3.54 | 13.89 | 26.07 | 9.0 |
| 74 | L15 × T4 | MGUH-74 | 7.04 | 4.60 | 9.34 | 10.37 | 4.45 | 14.82 | 26.46 | 10.0 |
| 75 | L15 × T5 | MGUH-75 | 10.26 | 5.84 | 13.18 | 10.47 | 4.98 | 15.45 | 31.54 | 7.7 |
| Mean | | | 8.02 | 4.69 | 10.37 | 11.22 | 5.25 | 16.47 | 29.18 | 8.9 |
| 76 | Check1 | CoMH08-292 | 2.65 | 1.23 | 3.27 | 19.43 | 12.23 | 31.66 | 35.54 | 10.4 |
| 77 | Check2 | DHM-121 | 2.07 | 1.84 | 2.99 | 19.95 | 12.15 | 32.09 | 36.00 | 8.6 |
| Mean | | | 2.36 | 1.53 | 3.13 | 19.69 | 12.19 | 31.88 | 35.77 | 9.5 |
| 78 | Check3 | Pusa HQPM-5 Improved | 9.98 | 3.72 | 11.85 | 11.97 | 4.91 | 16.87 | 30.58 | 8.7 |
| 79 | Check4 | Pusa HQPM-7 Improved | 10.47 | 3.97 | 12.45 | 9.08 | 6.34 | 15.43 | 29.86 | 8.5 |
| 80 | Check5 | Pusa Vivek QPM-9 Improved | 9.74 | 4.18 | 11.83 | 10.63 | 4.42 | 15.05 | 28.97 | 7.2 |
| Mean | | | 10.06 | 3.96 | 12.04 | 10.56 | 5.22 | 15.78 | 29.80 | 8.1 |
| CD at 5% | | | 0.48 | 0.33 | 0.50 | 0.86 | 0.46 | 1.04 | 1.23 | 0.8 |

BC: β- Carotene, BCX: β-Cryptoxanthin, ProA: Provitamin A, LUT: Lutein, ZEA: Zeaxanthin, Non-ProA: Non-Provitamin A, TC: Total carotenoids, YLD: Yield
