# Supplementary material for: Development of sub-tropically adapted diverse provitamin-A rich maize inbreds through marker-assisted pedigree selection, their characterization and utilization in hybrid breeding
Source: PLoS One. 2021 Feb 4;16(2):e0245497. doi: 10.1371/journal.pone.0245497 (PMC7861415; doi:10.1371/journal.pone.0245497)
Supplement: S5 Table — *Significant at p = 0.05; **Significant at p = 0.01, BC: β- Carotene, BCX: β-Cryptoxanthin, ProA: Provitamin-A, LUT: Lutein, ZEA: Zeaxanthin, Non-ProA: Non-Provitamin-A, TC: Total carotenoids, SE: Standard Error. (DOCX) [file pone.0245497.s005.docx]

**Table S5.** **GCA effects for different carotenoids of 20 parental inbreds across three locations.**

| **Lines** | | | **BC** | **BCX** | **ProA** | **LUT** | **ZEA** | **Non-proA** | **TC** |
| --- | --- | --- | --- | --- | --- | --- | --- | --- | --- |
| L1 | MGU-PVMAS-5 | | 0.47 ** | 0.35 ** | 0.64 ** | -1.01 ** | -1.27 ** | -2.29 ** | -1.58 ** |
| L2 | MGU-PVMAS-6 | | -1.12 ** | -0.80 ** | -1.52 ** | -0.33 * | -0.59 ** | -0.92 ** | -2.72 ** |
| L3 | MGU-PVMAS-7 | | 0.07 | 0.45 ** | 0.30 ** | -0.66 ** | -0.34 ** | -1.00 ** | -0.67 ** |
| L4 | MGU-PVMAS-8 | | 0.30 ** | 0.09 | 0.35 ** | 0.17 | 0.52 ** | 0.69 ** | 1.20 ** |
| L5 | MGU-PVMAS-9 | | -0.25 ** | -0.31 ** | -0.41 ** | 0.00 | -0.49 ** | -0.50 ** | -1.21 ** |
| L6 | MGU-PVMAS-10 | | 0.83 ** | 0.38 ** | 1.02 ** | -0.25 | 0.00 | -0.24 | 0.91 ** |
| L7 | MGU-PVMAS-11 | | 0.34 ** | 0.36 ** | 0.52 ** | 0.33 * | -0.09 | 0.24 | 1.05 ** |
| L8 | MGU-PVMAS-12 | | -1.04 ** | -0.42 ** | -1.25 ** | 0.74 ** | 0.72 ** | 1.46 ** | -0.03 |
| L9 | MGU-PVMAS-13 | | 0.06 | 0.05 | 0.08 | 0.66 ** | -0.34 ** | 0.32 | 0.55 ** |
| L10 | MGU-PVMAS-14 | | 0.30 ** | 0.052 | 0.32 ** | 1.27 ** | 0.87 ** | 2.14 ** | 2.17 ** |
| L11 | MGU-PVMAS-15 | | 0.42 ** | 0.069 | 0.45 ** | -0.31 * | 0.16 * | -0.15 | 0.45 * |
| L12 | MGU-PVMAS-1 | | 0.16 * | 0.79 ** | 0.55 ** | -0.93 ** | -0.40 ** | -1.33 ** | -0.27 |
| L13 | MGU-PVMAS-2 | | -0.42 ** | -1.04 ** | -0.94 ** | 1.07 ** | 1.83 ** | 2.90 ** | 1.35 ** |
| L14 | MGU-PVMAS-3 | | -0.20 ** | -0.40 ** | -0.40 ** | -0.05 | -0.07 | -0.13 | -0.60 ** |
| L15 | MGU-PVMAS-4 | | 0.09 | 0.39 ** | 0.28 ** | -0.70 ** | -0.50 ** | -1.20 ** | -0.60 ** |
| SE | | | 0.07 | 0.05 | 0.08 | 0.14 | 0.08 | 0.17 | 0.20 |
| Testers | | |  |  |  |  |  |  |  |
| T1 | | PMI-PV-9 | -0.55 ** | -0.56 ** | -0.83 ** | 1.39 ** | -0.30 ** | 1.11 ** | 0.12 |
| T2 | | PMI-PV-5 | 1.02 ** | 0.50 ** | 1.27 ** | -0.96 ** | -0.70 ** | -1.65 ** | -0.15 |
| T3 | | PMI-PV-6 | 0.20 ** | 0.28 ** | -0.06 | -0.64 ** | -0.08 | -0.72 ** | -0.59 ** |
| T4 | | PMI-PV-7 | -0.35 ** | 0.32 ** | -0.19 ** | -1.23 ** | -1.04 ** | -2.27 ** | -2.25 ** |
| T5 | | HP465-41 | 0.07 | -0.55 ** | -0.20 ** | 1.44 ** | 2.10 ** | 3.54 ** | 2.87 ** |
| SE | | | 0.04 | 0.03 | 0.05 | 0.08 | 0.04 | 0.10 | 0.12 |

*Significant at p = 0.05; **Significant at p = 0.01, BC: β- Carotene, BCX: β-Cryptoxanthin, ProA: Provitamin-A,

LUT: Lutein, ZEA: Zeaxanthin, Non-ProA: Non-Provitamin-A, TC: Total carotenoids, SE: Standard Error
