# Supplementary material for: Development of sub-tropically adapted diverse provitamin-A rich maize inbreds through marker-assisted pedigree selection, their characterization and utilization in hybrid breeding
Source: PLoS One. 2021 Feb 4;16(2):e0245497. doi: 10.1371/journal.pone.0245497 (PMC7861415; doi:10.1371/journal.pone.0245497)
Supplement: S6 Table — *Significant at p = 0.05; **Significant at p = 0.01. (DOCX) [file pone.0245497.s006.docx]

**Table S6. Percent economic heterosis of experimental hybrids over commercial checks across the locations.**

| **S No.** | **L × T**  **Code** | **Hybrids** | **% Economic heterosis over** | | | | |
| --- | --- | --- | --- | --- | --- | --- | --- |
|  |  |  | **CoMH-08-292** | **DHM-121** | **Pusa HQPM5 Improved** | **Pusa QPM7 Improved** | **Pusa Vivek QPM9 Improved** |
| 1 | L1 × T1 | MGUH-1 | -13.80 ** | 3.77 | 3.42 | 5.33 | 23.90 ** |
| 2 | L1 × T2 | MGUH-2 | -6.33 | 12.76 ** | 12.38 ** | 14.46 ** | 34.64 ** |
| 3 | L1 × T3 | MGUH-3 | -15.10 ** | 2.21 | 1.87 | 3.75 | 22.04 ** |
| 4 | L1 × T4 | MGUH-4 | -20.07 ** | -3.77 | -4.10 | -2.32 | 14.90 ** |
| 5 | L1 × T5 | MGUH-5 | -12.39 ** | 5.47 | 5.11 | 7.06 | 25.93 ** |
| 6 | L2 × T1 | MGUH-6 | -26.12 ** | -11.06 * | -11.36 * | -9.71 * | 6.20 |
| 7 | L2 × T2 | MGUH-7 | -18.69 ** | -2.12 | -2.44 | -0.64 | 16.87 ** |
| 8 | L2 × T3 | MGUH-8 | -18.08 ** | -1.38 | -1.71 | 0.11 | 17.76 ** |
| 9 | L2 × T4 | MGUH-9 | -8.34 * | 10.34 * | 9.97 * | 12.00 * | 31.75 ** |
| 10 | L2 × T5 | MGUH-10 | 0.41 | 20.88 ** | 20.47 ** | 22.70 ** | 44.33 ** |
| 11 | L3 × T1 | MGUH-11 | -16.11 ** | 0.99 | 0.65 | 2.51 | 20.58 ** |
| 12 | L3 × T2 | MGUH-12 | -19.30 ** | -2.85 | -3.17 | -1.38 | 16.00 ** |
| 13 | L3 × T3 | MGUH-13 | -11.23 ** | 6.87 | 6.51 | 8.48 | 27.60 ** |
| 14 | L3 × T4 | MGUH-14 | -26.41 ** | -11.41 * | -11.70 * | -10.07 * | 5.78 |
| 15 | L3 × T5 | MGUH-15 | 9.71 ** | 32.08 ** | 31.63 ** | 34.07 ** | 57.70 ** |
| 16 | L4 × T1 | MGUH-16 | -17.17 ** | -0.29 | -0.63 | 1.21 | 19.05 ** |
| 17 | L4 × T2 | MGUH-17 | -5.76 | 13.44 ** | 13.06 ** | 15.16 ** | 35.45 ** |
| 18 | L4 × T3 | MGUH-18 | -8.90 * | 9.67 * | 9.30 * | 11.32 * | 30.95 ** |
| 19 | L4 × T4 | MGUH-19 | -3.86 | 15.73 ** | 15.34 ** | 17.48 ** | 38.18 ** |
| 20 | L4 × T5 | MGUH-20 | -8.04 * | 10.70 * | 10.33 * | 12.38 ** | 32.18 ** |
| 21 | L5 × T1 | MGUH-21 | -11.81 ** | 6.17 | 5.81 | 7.77 | 26.76 ** |
| 22 | L5 × T2 | MGUH-22 | -3.39 | 16.30 ** | 15.91 ** | 18.06 ** | 38.86 ** |
| 23 | L5 × T3 | MGUH-23 | -3.83 | 15.78 ** | 15.39 ** | 17.52 ** | 38.24 ** |
| 24 | L5 × T4 | MGUH-24 | -5.85 | 13.34 ** | 12.96 ** | 15.06 ** | 35.33 ** |
| 25 | L5 × T5 | MGUH-25 | -3.10 | 16.66 ** | 16.27 ** | 18.42 ** | 39.29 |
| 26 | L6 × T1 | MGUH-26 | -28.66 ** | -14.11 ** | -14.40 ** | -12.82 ** | 2.55 |
| 27 | L6 × T2 | MGUH-27 | -30.17 ** | -15.94 ** | -16.22 ** | -14.67 ** | 0.37 |
| 28 | L6 × T3 | MGUH-28 | -14.95 ** | 2.39 | 2.05 | 3.94 | 22.26 ** |
| 29 | L6 × T4 | MGUH-29 | -19.48 ** | -3.07 | -3.40 | -1.61 | 15.73 ** |
| 30 | L6 × T5 | MGUH-30 | -8.87 ** | 9.70 * | 9.34 * | 11.36 * | 30.99 ** |
| 31 | L7 × T1 | MGUH-31 | -17.54 ** | -0.73 | -1.06 | 0.77 | 18.53 ** |
| 32 | L7 × T2 | MGUH-32 | -23.68 ** | -8.13 | -8.44 | -6.74 | 9.70 |
| 33 | L7 × T3 | MGUH-33 | -20.89 ** | -4.77 | -5.09 | -3.33 | 13.71 * |
| 34 | L7 × T4 | MGUH-34 | -18.73 ** | -2.17 | -2.50 | -0.69 | 16.81 ** |
| 35 | L7 × T5 | MGUH-35 | -13.29 ** | 4.38 | 4.03 | 5.96 | 24.63 ** |
| 36 | L8 × T1 | MGUH-36 | -22.39 ** | -6.57 | -6.88 | -5.16 | 11.56 * |
| 37 | L8 × T2 | MGUH-37 | -12.82 ** | 4.95 | 4.60 | 6.53 | 25.31 ** |
| 38 | L8 × T3 | MGUH-38 | -18.86 ** | -2.33 | -2.65 | -0.85 | 16.62 ** |
| 39 | L8 × T4 | MGUH-39 | -16.52 ** | 0.50 | 0.16 | 2.01 | 19.99 ** |
| 40 | L8 × T5 | MGUH-40 | -15.67 ** | 1.52 | 1.18 | 3.05 | 21.22 ** |
| 41 | L9 × T1 | MGUH-41 | -33.10 ** | -19.47 ** | -19.74 ** | -18.25 ** | -3.85 |
| 42 | L9 × T2 | MGUH-42 | -6.28 | 12.82 ** | 12.44 ** | 14.52 ** | 34.71 ** |
| 43 | L9 × T3 | MGUH-43 | -23.31 ** | -7.68 | -7.99 | -6.29 | 10.23 |
| 44 | L9 × T4 | MGUH-44 | -10.37 ** | 7.91 | 7.54 | 9.53 * | 28.84 ** |
| 45 | L9 × T5 | MGUH-45 | -11.18 ** | 6.92 | 6.56 | 8.53 | 27.66 ** |
| 46 | L10 × T1 | MGUH-46 | -22.84 ** | -7.11 | -7.43 | -5.71 | 10.91 * |
| 47 | L10 × T2 | MGUH-47 | -46.49 ** | -35.58 ** | -35.80 ** | -34.61 ** | -23.08 ** |
| 48 | L10 × T3 | MGUH-48 | -18.13 ** | -1.45 | -1.78 | 0.04 | 17.67 ** |
| 49 | L10 × T4 | MGUH-49 | -3.68 | 15.95 ** | 15.56 ** | 17.70 ** | 38.44 ** |
| 50 | L10 × T5 | MGUH-50 | -0.28 | 20.04 ** | 19.64 ** | 21.86 ** | 43.33 ** |
| 51 | L11 × T1 | MGUH-51 | -22.63 ** | -6.86 | -7.17 | -5.46 | 11.21 * |
| 52 | L11 × T2 | MGUH-52 | -23.80 ** | -8.27 | -8.58 | -6.89 | 9.52 |
| 53 | L11 × T3 | MGUH-53 | -13.20 ** | 4.50 | 4.15 | 6.08 | 24.77 ** |
| 54 | L11 × T4 | MGUH-54 | -1.51 | 18.57 ** | 18.17 ** | 20.36 ** | 41.57 ** |
| 55 | L11 × T5 | MGUH-55 | -0.79 | 19.43 ** | 19.03 ** | 21.24 ** | 42.61 ** |
| 56 | L12 × T1 | MGUH-56 | -20.64 ** | -4.47 | -4.79 | -3.03 | 14.06 * |
| 57 | L12 × T2 | MGUH-57 | -22.25 ** | -6.40 | -6.71 | -4.99 | 11.76 * |
| 58 | L12 × T3 | MGUH-58 | -13.04 ** | 4.68 | 4.33 | 6.26 | 24.99 ** |
| 59 | L12 × T4 | MGUH-59 | -10.19 ** | 8.12 | 7.76 | 9.75 * | 29.10 ** |
| 60 | L12 × T5 | MGUH-60 | -7.45 * | 11.42 * | 11.04 * | 13.10 ** | 33.03 ** |
| 61 | L13 × T1 | MGUH-61 | -25.60 ** | -10.44 * | -10.74 | -9.09 | 6.94 |
| 62 | L13 × T2 | MGUH-62 | -9.95 ** | 8.40 | 8.04 | 10.04 * | 29.43 ** |
| 63 | L13 × T3 | MGUH-63 | -8.23 * | 10.48 * | 10.11 * | 12.15 ** | 31.91 ** |
| 64 | L13 × T4 | MGUH-64 | -4.66 | 14.77 ** | 14.38 ** | 16.50 ** | 37.03 ** |
| 65 | L13 × T5 | MGUH-65 | -3.18 | 16.56 ** | 16.17 ** | 18.32 ** | 39.17 ** |
| 66 | L14 × T1 | MGUH-66 | -12.47 ** | 5.38 | 5.02 | 6.97 | 25.82 ** |
| 67 | L14 × T2 | MGUH-67 | -15.89 ** | 1.25 | 0.91 | 2.78 | 20.89 ** |
| 68 | L14 × T3 | MGUH-68 | -13.79 ** | 3.79 | 3.44 | 5.35 | 23.92 ** |
| 69 | L14 × T4 | MGUH-69 | 1.49 | 22.18 ** | 21.77 ** | 24.02 ** | 45.88 ** |
| 70 | L14 × T5 | MGUH-70 | -26.69 ** | -11.74 * | -12.04 ** | -10.41 * | 5.38 |
| 71 | L15 × T1 | MGUH-71 | -20.46 ** | -4.25 | -4.57 | -2.80 | 14.33 ** |
| 72 | L15 × T2 | MGUH-72 | -2.56 ** | 17.30 ** | 16.91 ** | 19.07 ** | 40.06 ** |
| 73 | L15 × T3 | MGUH-73 | -12.80 ** | 4.98 | 4.63 | 6.56 | 25.35 ** |
| 74 | L15 × T4 | MGUH-74 | -2.82 | 16.99 ** | 16.60 ** | 18.76 ** | 39.69 ** |
| 75 | L15 × T5 | MGUH-75 | -25.41 ** | -10.21 * | -10.51 * | -8.85 | 7.22 |

*Significant at p = 0.05; **Significant at p = 0.01
