# Supplementary figures and images for: Development of sub-tropically adapted diverse provitamin-A rich maize inbreds through marker-assisted pedigree selection, their characterization and utilization in hybrid breeding
Source: PLoS One. 2021 Feb 4;16(2):e0245497. doi: 10.1371/journal.pone.0245497 (PMC7861415; doi:10.1371/journal.pone.0245497)

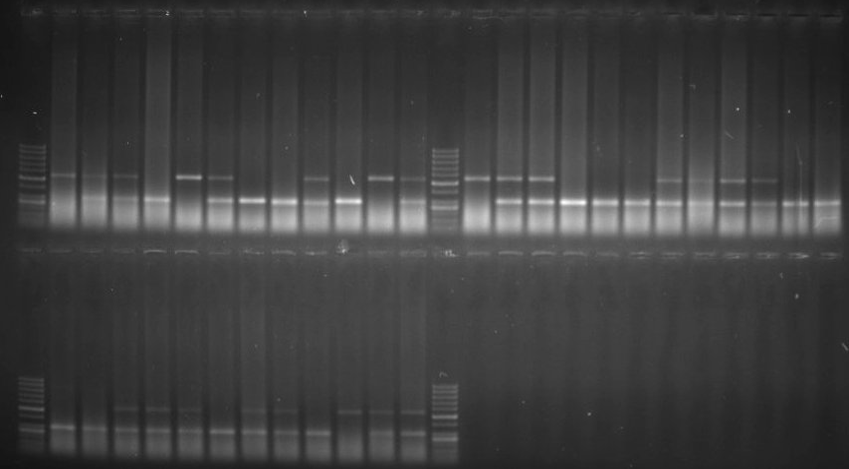


S1 file. Segregation of favourable (543 bp) and unfavourable (296 bp) alleles of *crtRB1* in F2 populations

Supplement: S1 File — (DOCX) [file pone.0245497.s007.docx]
